# The HCV Envelope Glycoprotein Down-Modulates NF-κB Signalling and Associates With Stimulation of the Host Endoplasmic Reticulum Stress Pathway

**DOI:** 10.3389/fimmu.2022.831695

**Published:** 2022-03-15

**Authors:** Lindsay G. A. McKay, Jordan Thomas, Wejdan Albalawi, Antoine Fattaccioli, Marc Dieu, Alessandra Ruggiero, Jane A. McKeating, Jonathan K. Ball, Alexander W. Tarr, Patricia Renard, Georgios Pollakis, William A. Paxton

**Affiliations:** ^1^ Department of Clinical Infection, Microbiology and Immunology, Institute of Veterinary and Ecological Sciences, University of Liverpool, Liverpool, United Kingdom; ^2^ Laboratory of Biochemistry and Cell Biology (URBC), Namur Research Institute for Life Sciences (NARILIS), University of Namur (UNamur), Namur, Belgium; ^3^ MaSUN, Mass Spectrometry Facility, University of Namur (UNamur), Namur, Belgium; ^4^ Nuffield Department of Medicine, University of Oxford, Oxford, United Kingdom; ^5^ Wolfson Centre for Global Virus Research and School of Life Sciences, University of Nottingham, Nottingham, United Kingdom

**Keywords:** immunity, HIV-LTR, HCV, NF-κB, endoplasmic reticulum stress

## Abstract

Following acute HCV infection, the virus establishes a chronic disease in the majority of patients whilst few individuals clear the infection spontaneously. The precise mechanisms that determine chronic HCV infection or spontaneous clearance are not completely understood but are proposed to be driven by host and viral genetic factors as well as HCV encoded immunomodulatory proteins. Using the HIV-1 LTR as a tool to measure NF-κB activity, we identified that the HCV E1E2 glycoproteins and more so the E2 protein down-modulates HIV-1 LTR activation in 293T, TZM-bl and the more physiologically relevant Huh7 liver derived cell line. We demonstrate this effect is specifically mediated through inhibiting NF-κB binding to the LTR and show that this effect was conserved for all HCV genotypes tested. Transcriptomic analysis of 293T cells expressing the HCV glycoproteins identified E1E2 mediated stimulation of the endoplasmic reticulum (ER) stress response pathway and upregulation of stress response genes such as ATF3. Through shRNA mediated inhibition of ATF3, one of the components, we observed that E1E2 mediated inhibitory effects on HIV-1 LTR activity was alleviated. Our *in vitro* studies demonstrate that HCV Env glycoprotein activates host ER Stress Pathways known to inhibit NF-κB activity. This has potential implications for understanding HCV induced immune activation as well as oncogenesis.

## Introduction

Hepatitis C Virus (HCV) is a member of the *Flaviviridae* family which infects approximately 58 million people worldwide and can mediate severe hepatic injury and progressive liver fibrosis through stimulating persistent inflammation and oxidative stress (1–5). Approximately 50-80% of HCV positive individuals fail to resolve the infection whilst the remaining patients spontaneously clear virus within 6 months, though the mechanisms that determine either of these outcomes are incompletely understood (6–9). Several factors are associated with increased likelihood of chronic HCV infection including a higher diversity of HCV quasispecies (10), ethnicity of the host (9, 11, 12) and co-infection with Human Immunodeficiency Virus Type 1 (HIV-1) (13–15). Conversely, factors such as sexual transmission (16), Interleukin 28-B (IL28B) genotype CC (17–19), several Human Leukocyte Antigen (HLA) types including HLA-B*57, female sex and the strength of HCV specific T cell responses are associated with increased likelihood of spontaneous clearance (20, 21).

HCV encoded immunomodulatory factors have been proposed to influence the establishment of chronic infection by interfering with intracellular innate immunity pathways (22). For example, it has been suggested that the HCV core protein modulates cellular immune responses *via* downregulation of Nuclear Factor Kappa-Light-Chain-Enhancer of Activated B Cells (NF-κB) activation (23–25), inhibition of Interleukin 2 (IL-2) production (26) and suppression of CD8+ and CD4+ T cell responses (27, 28). Similarly, the HCV NS2 and NS3/4A proteins down-modulate early innate immune responses by inhibiting key steps in type 1 Interferon (IFN) signalling pathways *in vitro* (29–32) and *in vivo* (33). Additionally, infection with HCV may down-modulate cellular immune responses indirectly, through upregulation of stress responses. Infection with HCV *in vitro* stimulates endoplasmic reticulum (ER) stress and leads to the induction of the Unfolded Protein Response (UPR) and autophagy. These cryoprotective responses function to resolve ER stress by reducing protein synthesis and increasing the protein processing capacity of the infected cell (34–36). Nevertheless, upregulation of ER stress has been proposed to promote HCV replication (37–39) and also down-modulate innate immune signalling in the infected cell (36), providing a favourable landscape for HCV replication and potentially aiding the establishment of chronic infection. Several reports have identified that individual proteins of HCV are potent activators of the UPR independent of HCV replication, including the envelope (Env) glycoproteins E1 and E2 (40–42). Both E1 and E2 are highly glycosylated type 1 transmembrane glycoproteins which use membrane anchored C-terminal tails to remain tethered in the endoplasmic reticulum (ER) prior to a series of protein maturation events (43). Currently, there is limited understanding regarding the molecular mechanisms through which HCV Env glycoproteins may modulate cellular immune responses and contribute to HCV mediated immune regulation.

Here we studied the interaction between the HCV Env glycoprotein and its effects on modulating HIV-1 Long Terminal Repeat (LTR) activation as a tool to measure the effects of these proteins on NF-κB signalling in 293T, TZM-bl and the more physiologically relevant Huh7 cell line. Using plasmids that express HIV-1 or luciferase under the control of the HIV-1 LTR, we investigated the ability of HCV Env glycoprotein to modulate cellular transcription pathways that may down-modulate immune signalling and promote the establishment of chronic HCV infection.

## Materials and Methods

### Cell Culture

Cell lines 293T, Huh7 and TZM-bl were cultured in DMEM. All media was supplemented with 10% heat inactivated FBS (non-US origin, Sigma-Aldrich) and 1% L-glutamine (Gibco) and cells grown at 5% CO_2_/37°C. Similarly, J-Lat 10.6 cells were maintained in RPMI-1640 supplemented with 10% heat inactivated FBS (non-US origin, Sigma-Aldrich) and grown at 5% CO_2_/37°C.

### Cell Viability Assays

For measurement of cell viability following transfection with shRNA and viral envelope expression plasmids, 293T cells were seeded in a 12 well plate (7.5x10^4^ per well). At 24 h post seeding cells were transfected with 150 ng of ATF3 shRNA plasmids or the scrambled shRNA or 12 ng of HCV E1E2, HCV SE2, HIV-1 JRFL (R5), HIV-1 LAI (X4), EBOV Env expressed plasmids using the polyethyleneimine (PEI) protocol. At 73 h post transfection cells were washed with PBS and 1:1 mixture of the cell suspension and 0.4% trypan blue solution was prepared. Cell viability was determined using automated cell counter. Similarly, viable cell counts for 293T and TZM-bl cells stably transfected with E1E2 were measured *via* 0.4% trypan blue solution and compared with the non-transfected cells.

### Plasmid Preparation

Heat shock transformation followed by purification with Qiagen miniprep or maxiprep kits were used. In short, 2 µL plasmid stock (varying from 10 to 100 ng) was added to One Shot Top10 bacteria (ThermoFisher), mixed and incubated on ice for 30 min then incubated at 42°C for 30 s followed by 30 s on ice. 0.5 mL Super Optimal broth with Catabolite repression (SOC) media (ThermoFisher) was added and incubated for 1 h at 37°C with 180 rpm shaking before being plated on antibiotic selective agar plates overnight. Colonies were picked and grown in 5 mL Brain Heart Infuction (BHI)/ampicillin (100 µg/ml) culture (6 h - 16 h, 180 rpm at 37°C) for miniprep isolation (Qiagen), which was used to check plasmids before larger maxipreps were performed. For maxipreps (Qiagen), 250 mL BHI/ampicillin cultures were used and grown overnight using the same conditions as stated above.

### Generation of Pseudo-Typed Viral Particles

HIV-1 JRFL (R5), HIV-1 LAI (X4), EBOV GP (Ebola) and HCV E1E2 Env (H77 isolate genotype 1a) were transfected into 293T cells in 10 cm dishes using the PEI protocol. For all transfections, PEI was diluted to 0.08 µg/µl in OptiMEM (Gibco) and all cells were incubated in 5% CO_2_/37°C. For pseudo-typed virus particle production, the ΔEnv backbone pNL4-3-luc was used at 2,000 ng per condition as well as either 2000 ng of the HIV-1 Env expression plasmid, 285 ng of the Ebola Env plasmid or 200 ng of HCV Env E1E2 expression plasmids. In all cases, plasmids were added to 300 µl of Opti-MEM and this was then added to 300 µl of diluted PEI solution, mixed and incubated at rt for 20 min. Media was removed from 10 cm dishes and replaced with 1 ml Opti-MEM, and following incubation, transfection mix was added to cells dropwise and mixed and incubated under cell culture conditions for 6 h. Following this incubation, transfection media was replaced with DMEM.

### Infection with Pseudo-Typed Viral Particles

Infections were performed on TZM-bl, Huh7 and 293T cell lines. Viral stocks were quantified *via* capsid-p24 standardised input. Target cells were seeded in 96 well plates (1.5x10^4^ cells/well) 24 h prior to infection. Media was removed from the plate before 50 µL required media was added, followed by 100 µL of virus stock. After 6 h of incubation an additional 100 µL of appropriate cell media was added to each well and 48 h later cells were lysed and luciferase activity was quantified using BMG Labtech FLUOstar Omega with 100 µl luciferase substrate (Promega) injected per well.

### Viral Quantification (p24 Capsid Assay)

The p24 assay was performed using recombinant p24 standard, p24 coating antibody (polyclonal sheep anti-HIV-1 p24 gag), secondary conjugate (alkaline phosphatase conjugate of mouse monoclonal anti-HIV-1 p24) from Aalto Bio Reagents Ltd and according to their protocol. Detection was performed using ELISA-Light Immunoassay System with CSP and Sapphire-I Substrate/Enhancer (ThermoFisher). All samples were diluted using 0.1% Empigen/TBS. Luminescence from ELISA plates was measured using a BMG Labtech FLUOstar Omega luminometer with a reading interval of 1 sec/well.

### LTR, sE2 and E1E2 Transfection of Cell Lines

293T cells were seeded on 96 well plates (1.5x10^4^ cells/well). When confluent, media was removed and 50 µL of Opti-MEM added to each well. Two µL Opti-MEM added per well with the following plasmid quantities: 12, 6 or 1 ng E1E2 or sE2 plasmid (nanogram quantity equalised with pCDNA between conditions), 6 ng LTR and 1 ng Tat. PEI was diluted to 0.14 µg/µl in 200 µL Opti-MEM and 2 µL of the diluted PEI was added to the plasmid mix. The plasmid/PEI mix was incubated at rt for 30 min before being added to cells and incubating for 6 h at 5% CO_2_/37°C after which Opti-MEM/plasmid mix was removed and 250 µL DMEM added. Plates were measured for luciferase activity 48 h after transfection as described above. The following genotypes of HCV E1E2 Env were used – 1a, 1b, 2b, 3, 4, 5 and 6 (44, 45). HCV sE2 plasmids containing the gene fragment for the soluble ectodomain of the HCV E2 protein (aa363-661, referenced to the HCV strain H77 polyprotein) were PCR amplified from full length E1/E2 plasmids using genotype-specific primers [primer sequences described previously (45)] and Expand High-Fidelity Polymerase PCR System (Roche). PCR products were sub-cloned into the pCDNA plasmid and possessed an in-frame C-terminal 6xHis tag sequence. The following genotypes of HCV sE2 envelope were used – 1a, 1b, 2b, 4, 5 and 6. No corresponding sE2 genotype 3a plasmid was available.

### Western Blot Analysis

Activating Transcription Factor 3 (ATF3) protein expression was determined in 293T cells transfected with ATF3 shRNA expression plasmids, HCV E1E2 Env and scrambled shRNA and pCDNA controls using western blot. Following transfection, cells were washed in ice cold dPBS and lysed with radioimmunoprecipitation assays (RIPA) buffer that included a 1% protease inhibitor cocktail (Thermo Scientific) and the protein concentration was subsequently quantified by the Bradford assay using the Protein Assay kit (Bio-Rad). 30 µg of cell lysates were mixed with 4x NuPage LDS Sample Buffer and 10x NuPage reducing agent, incubated at 72°C for 10 mins and loaded onto polyacrylamide gels (NuPAGE 12% Bis-Tris Gels; ThermoFisher), and electrophoresed at 120 V for 60 min.

Separated proteins were transferred to iBlot™ 2 PVDF Mini Stacks membranes (ThermoFisher) employing the iBlot 2 Dry Blotting system. The membranes were then blocked using the iBind solution kit (ThermoFisher) and incubated with primary and secondary antibodies using the iBind device (Invitrogen, USA) for 3 h. Antibodies used in this study included rabbit anti-ATF3 (1:250, Abcam), rabbit anti-β-actin (1:250, Abcam) and horseradish peroxidase (HRP) conjugated secondary antibody (anti-rabbit IgG; 1:1000; Bio-Rad). Protein bands were visualized using Pierce™ ECL Plus Western Blotting Substrate (ThermoFisher).

### Stable Cell Line Production

TZM-bl cells were seeded in a 12 well plate (7.5x10^4^ cells/well). At 24 h post seeding cells were transfected with either an E1E2 or sE2 expression plasmid containing a V5 tagged genotype 1A E1E2 or sE2 gene (expressing neomycin resistance) using the PEI protocol. Briefly, the transfection mix was prepared by diluting PEI to 0.08 µg/µl in 50 µL Opti-MEM to which 50 ng of envelope plasmid was added. This mix was incubated for 30 min at rt before being added to the well and incubated for 6 h in cell culture conditions, after which Opti-MEM/plasmid mix was removed and 1 mL DMEM was added. At 48 h post transfection media was refreshed using antibiotic selection (optimised as 400 µg/ml G418). Resistant cells were expanded before being tested for plasmid expression *via* FACS analysis (V5 directed Ab) and monitored periodically. TZM-bl-E1E2 and TZM-bl-sE2 cell lines were generated (E1E2: UKN1A20.8 EU155192. sE2: soluble E2_661 1A20.8.4). E1E2 and sE2 expression was confirmed *via* FACS staining of cells. In short, 2.5x10^5^ cells were harvested 48 hours post transfection and were resuspended in 100 µl 4% paraformaldehyde solution for 20 minutes at 4°C. Cells were then washed twice in staining buffer (0.2 µm pore filtered PBS/1% heat inactivated FBS) and resuspended in fixation buffer (PBS/1% heat inactivated FBS/0.5% saponin) for 15 mins at room temperature. Cells were then resuspended in 50 µl fixation buffer containing the primary antibody (AP33 primary antibody at a 1:10 dilution) for 30 minutes at 4°C in the dark. Cells were washed with fixation buffer twice before being resuspended in 20 µl fixation buffer containing the secondary antibody (anti-mouse goat PE conjugate - 1:5 dilution), for 30 mins at 4°C in the dark. Cells were then washed and resuspended in 200 µl staining buffer at 4°C in the dark until FACS analysis. The AP33 antibody recognises E2 epitopes between aa412-423.

### Nuclear Extraction

TZM-bl and TZM-bl-E1E2 cells were grown to required confluency prior to extraction. Cells were washed with 10 mL cold PBS, followed by 10 mL cold PBS supplemented with 5 mM NaF and 1 mM Na_2_MoO_4_. 10 mL HB 1 x (2x: PBS supplemented with 40 mM HEPES, 10 mM NaF, 2 mM Na_2_MoO_4_ and 0.2 mM EDTA) was then added for 5 mins before being removed and 0.6 mL lysis buffer (10 mL HB 2x supplemented with 400 µL 10% NP-40 and 9.6 mL H_2_0) added for a further 5 min incubation. The cells were transferred to an Eppendorf microcentrifuge tube and centrifuged at 13,000 rpm for 60 s. The supernatant was removed and the pellet resuspended in 50 µL resuspension buffer (10 mL HB 2x, 4 mL 97% glycerol and 6 mL H_2_O). 60 µL salt buffer (10 mL HB 2x, 4 mL 87% glycerol, 4 mL 4M NaCl and 2 mL H_2_0) was then added before the samples were incubated for 1 hr on a rotating wheel. 1 mL each resuspension buffer and salt buffer, supplemented with 40 µL PIC 25x (protease inhibitor cocktail, Sigma-Aldrich) and 100 µL Phospho Stop 10x (phosphatase inhibitor cocktail, Sigma-Aldrich) added prior to use. The samples were centrifuged at 13,000 rpm for 10 min before protein concentration quantified *via* the Bradford assay. Once quantified, the samples were aliquoted and stored at -80°C. All steps of this protocol were undertaken either on ice or at 4°C. Bradford quantification was performed using Quick Start Bradford Reagent (Bio-Rad).

### HIV-1-LTR Capture Probe Synthesis and Affinity Purification

The DNA capture probe production and affinity purification were performed as described (46). Briefly, 20 pmoles of a 226-bp-long desthiobiotinylated double-stranded oligonucleotide corresponding to a fragment of the HIV-1 5’ LTR (nt 229–455, where nt 1 is the start of the 5’ LTR U3 region) that contains 2 NF-κB binding sites was produced by polymerase chain reaction (PCR) and immobilized on 1 mg of streptavidin-coated magnetic beads. 1 mg of protein nuclear extract prepared as described above was incubated for 2 h with the DNA-coated beads. After washes, biotin was used to specifically elute the protein-DNA complexes, before protein digestion with trypsin. Excess of biotin was finally removed by an additional incubation in the presence of 600 µg of fresh streptavidin-coated magnetic beads.

### Liquid Chromatography/Tandem Mass Spectrometry Analysis and Protein Identification

Peptides were analysed using nano-LC-ESI-MS/MS maXis Impact UHR-TOF (Bruker, Bremen, Germany) coupled with a UPLC Dionex UltiMate 3000 (ThermoFisher). Digests were separated by reverse-phase liquid chromatography using a 75 µm X 250 mm reverse phase column (Acclaim PepMap 100 C18, ThermoFisher) in an Ultimate 3000 liquid chromatography system. Mobile phase A was 95% of 0.1% formic acid in water and 5% acetonitrile. Mobile phase B was 0.1% formic acid in acetonitrile. The runtime was 120 min. The digest (15 µL) was injected, and the organic content of the mobile phase was increased linearly from 4% B to 35% in 90 min and from 35% B to 90% B in 5 min. The column effluent was connected to a Captive Spray (Bruker). In survey scan, MS spectra were acquired for 0.5 s in the m/z range between 50 and 2200. The 10 most intense peptides ions 2+ or 3+ were sequenced. The collision-induced dissociation (CID) energy was automatically set according to mass to charge (m/z) ratio and charge state of the precursor ion. The MaXis and the Ultimate systems were piloted by Compass HyStar 3.2 (Bruker).

Peak lists were created using DataAnalysis 4.1 (Bruker) and saved as MGF file for use with ProteinScape 3.1 (Bruker) with Mascot 2.4 as search engine (Matrix Science). Enzyme specificity was set to trypsin, and the maximum number of missed cleavages per peptide was set at one. Carbamidomethylation was allowed as fixed modification, oxidation of methionine, Gln–pyro-Glu and Carbamidomethylation (N-term) were allowed as variable modification. Mass tolerance for monoisotopic peptide window was 7 ppm and MS/MS tolerance window was set to 0.05Da. The peak lists were searched against a subset from the taxonomy homo sapiens of the UNIPROT database (162267 entries). Scaffold software (Scaffold 4.8.4, Proteome Software) was used to validate MS/MS based peptide and protein identification. For each sample, two analyses were made and results coming from Mascot were merged before performing a second search with X!Tandem (The GPM, the gpm.org). Peptide identifications were accepted if above 75% probability to achieve an FDR less than 1% by the Scaffold local FDR algorithm. Protein identifications were accepted if above 11% probability to achieve an FDR less than 2% and contained at least 2 identified peptides. Proteins sharing significance peptide evidence were grouped into clusters. Spectral counting quantitative analysis was used to compare the samples. T-test with a significance level of p<0.05 and a normalization based on total spectra were applied to the results.

### Gene Expression PCR Detection

293T cells were plated o/n in six well plates (2.5x10^5^ cells per well). The media was replaced with 500 µL of Opti-MEM per well. 250 ng DNA was added to 300 µL Opti-MEM media and mixed with 300 µL 0.08 µg/µl PEI and incubated for 30 mins before added to the cells and incubated for 48 h. Total RNA was extracted using the RNeasy Mini Kit (Qiagen). Reverse transcription of total RNA was performed using the Superscript III cDNA kit (ThermoFisher). Quantitative RT-PCR measuring relative expression of NF-κB (SinoBilogical, HP 100039), RELA (SinoBilogical, HP100270), IFI16 (ThermoFisher, Hs06603201_cn) and RBMX (ThermoFisher, Hs02426405_cn) was performed using SYBR green master mix, 1 µL primers, 4 µL nuclease free water and 5 µL of cDNA in total reaction volume of 20 µL. GAPDH (SinoBilogical, HP100003) was used as a control housekeeping gene. Samples were amplified and detected using a GeneRoter (Qiagen). Reactions were prepared in triplicate for each sample. PCR reaction conditions: 50°C for 2 min, 95°C for 5 min, 95°C for 10 s, 60°C for 20 s, 72°C for 10 s and a total of 40 amplification cycles were performed with the final extension being 60°C for 5 min. The results were normalised to GAPDH expression.

### MinION RNA-Seq

293T cells were seeded at 4.8x10^5^ cells per well in a 6-well plate in 3.5 ml DMEM complete medium 24 h prior to transfection. On the day of transfection, culture medium was removed and replaced with 500 µl Opti-MEM. Transfection mix was prepared by diluting PEI to 0.08 µg/µl in 300 µl OptiMEM, to which 384 ng of HCV E1E2 glycoprotein expressing plasmid or control empty pCDNA vector was added and incubated at rt for 30 min. Transfection mix was added dropwise onto cells and incubated for 6 h in cell culture conditions. Following incubation, transfection mix was removed and replaced with 3.5 ml DMEM complete medium and incubated for 48 h. Cells were then washed in warm PBS and lysed using RLT lysis buffer (Qiagen) supplemented with β-mercaptoehtanol. RNA was purified from lysates using RNeasy Plus Minikit (Qiagen), according to manufacturer’s instructions and eluted in 35 µl nuclease free water. RNA quality and purity were assessed using a Nanodrop spectrophotometer and Agilent Bioanalyzer, which indicated that all samples had an RNA Integrity Number (RIN) >9.3 (min 9.4 and max 10.0). RNA was quantified using Qubit high sensitivity RNA fluorometer. PolyA+ mRNA was purified from 35 µg total RNA using Dynabeads mRNA purification kit (ThermoFisher) and eluted in 15 µl of nuclease free water. Libraries were prepared using 30 ng polyA+ mRNA according to the Oxford Nanopore SQK-PCS-109 protocol in conjunction with EXP-PBC-001 barcoding kit to allow multiplexing of flow cells. Briefly, reverse transcription was performed using Maxima H Minus Reverse Transcriptase (42°C for 90 min, 85°C for 5 min) and 5 µl of reverse transcription product was separated into 2x 50 µl reactions with Oxford Nanopore barcoded primers for amplification (95°C for 5 min and 12 cycles of 95°C for 15 sec, 62°C for 15 sec and 65°C for 8 min, with final elongation at 65°C for 8 min). Amplification products were pooled and purified using Ampure XP magnetic beads (Beckman Coulter) with a 0.45x ratio of beads to DNA volume. Three adapted libraries were sequenced per flow cell, using 100 ng of each library. Libraries derived from control pCDNA transfected cells and libraries derived from HCV E1E2 transfected cells were sequenced on the same flow cells to limit flow cell associated bias. Sequencing was performed using MIN-106 flow cells (R9.4.1 or R9.4 pores) on the Oxford Nanopore MinION sequencing platform. Run durations ranged from 12-24 h.

### Differential Gene Expression Analysis

MinION reads were basecalled using Oxford Nanpore’s Guppy Basecaller software (version 3.2.4) and reads with a q-score <7 were discarded during basecalling. Each sequencing library was demultiplexed and adapter sequences were trimmed using Porechop (https://github.com/rrwick/Porechop). To confirm expression of HCV E1E2 in transfected cells, libraries were mapped to a database of viral genomes using Kraken2 (47). Reads were then mapped to human genome HG38 (GCA_000001405.15) using Minimap2 (48) with parameters “–ax –splice -k14”. Mapped reads were assigned to genomic features using featureCounts (49). Normalisation of raw read counts was performed using the EdgeR “trimmed mean M-values (TMM)” method (50). Genes with read counts lower than a sufficient value were filtered out using the EdgeR function “filterByExpr” with the default parameters (50). Differential gene expression between E1E2 or pCDNA transfected cells was determined using the R package Limma, using the “voom” method with a cut off of p=0.05 (51, 52). clusterProfiler was used to determine the biological themes of differentially expressed genes (53). Gene set enrichment analysis was performed with 10,000 gene set permutations to identify possible pathways that may be affected by E1E2 (54).

### ATF3 shRNA Knock-Down

Three ATF3 directed shRNA and a scrambled expression plasmid (pSUPER) were a kind gift from Dr Martin Janz (Max Delbrueck Center for Molecular Medicine and Charité, University Hospital Berlin) (55). 293T cells were seeded in 24 well plates (1x10^5^ cells per well) 24 h prior to transfection. On day of transfection, medium was replaced with 100 µl Opti-MEM. Transfection mix was prepared by diluting PEI to 0.08 µg/µl in 50 µl Opti-MEM to which 300 ng of ATF3 shRNA plasmids or the scrambled shRNA or pCDNA control was added. The mix was incubated at rt for 30 min before adding to cells and incubated for 6 h after which the Opti-MEM/plasmid mix was removed and 1 ml DMEM added. After 48 h cells were re-transfected with 50 ng of LTR-Luc and 5 ng Tat expression plasmids, using the same conditions as described above. The plate was measured for luciferase activity 48 h after transfection. Similar was performed utilising a 96 well plate format (1.5x10^4^ cells per well) following the same procedures with reagent volumes and quantities adjusted accordingly.

### Statistical Analysis

Statistical analysis was performed using GraphPad Prism 8 software unless specified otherwise. For RNAseq, statistical analysis was performed using R packages Voom/Limma. Data were analysed using the Kruskal-Wallis one-way analysis of variance followed by using Dunn’s analysis to perform paired multiple comparisons. Ns – non-statistical. * P<0.05. ** P<0.01.

## Results

### HCV E1E2 Env Glycoprotein Down-Modulates HIV-1 Viral Production

**Figure 1:**
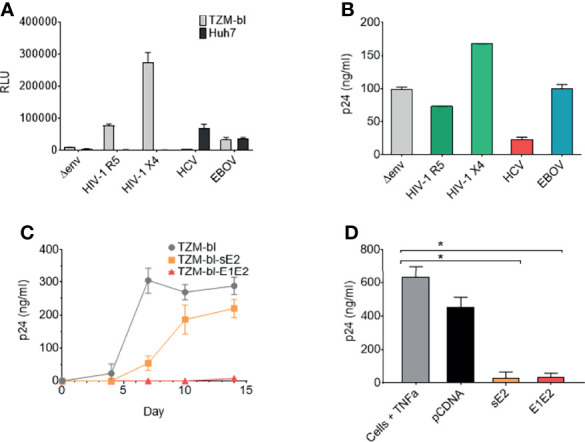
The HCV E1E2 Env and sE2 Env proteins can restrict HIV-1 replication, reduce HIV-1 infectious virus production and restrict proviral activation. **(A)** Pseudo-typed virus and infection of TZM-bl or Huh7 cell lines. ΔEnv backbone, JR-FL HIV-1 (CCR5 using) Env pseudo-typed virus, LAI HIV-1 Env (CXCR4 using) pseudo-typed virus, E1E2 HCV Env pseudo-typed virus and Ebola virus (EBOV) Env GP pseudo-typed viruses were infected onto TZM-bl and Huh7 cells using a standardised 10 ng (p24) of pseudo-typed virus input. Infection was quantified *via* luciferase readout (RLUs) (n=3). **(B)** Pseudo-typed virus quantification *via* p24 capsid ELISA (ng/mL) for each of the enveloped viruses with ΔEnv virus used as a control (n=2). **(C)** Replication curves of LAI-YFP (TCID50/ml 10,000 infectious titre) on three cell lines: TZM-bl, TZM-bl-E1E2 and TZM-bl-sE2. Replication was quantified *via* p24 capsid ELISA at four timepoints: day 4, 7, 10 and 14 post infection (n = 4). **(D)** The production of virus as quantified *via* p24 capsid ELISA at day 7 from J-Lat 10.6 cells post TNFα activation and transfection of cells with Env glycoproteins or pCDNA empty vector (n = 4). Kruskal-Wallis and Dunn’s test were used to analyse significance between the control cells and all other conditions. *P < 0.05. For all graphs mean is plotted and error bars represent standard deviation.

Pseudo-typed lentiviral particle systems are routinely utilised to study viral phenotypes (including HIV-1, HCV, Ebola virus and coronavisuses). Transfection of 293T producer cells with a plasmid expressing the HIV-1 viral backbone minus the Env gene (Δ*env*) in conjunction with a plasmid expressing viral Env proteins was performed. When producing pseudo-typed lentiviral particles expressing the HCV envelope E1E2 a virus stock was generated that infects liver derived Huh7 cells but not TZM-bl cells, indicating HCV E1E2 protein expression (**Figure 1A**). Through generating pseudo-typed viral stocks expressing variant Env proteins [HIV-1 (R5), HIV-1 (X4), HCV Env and Ebola virus glycoprotein (GP)] a consistent decrease in viral p24 production was observed for the HCV E1E2 stocks in comparison to other viruses (observed in >3 different experiments) (**Figure 1B**). We confirmed HCV E1E2 Env expression from analysing stably transfected TZM-bl cells with a plasmid expressing a tagged E1E2 molecule using FACS (**Supplementary Figure 1**). These findings identify that expression of the HCV E1E2 Env protein down-modulates pseudo-typed viral production in 293T producer cells.

### HCV E1E2 and sE2 Expression Can Reduce HIV-1 Replication and Activation

From the above observations, we hypothesised that specific expression of HCV E1E2 protein can down-modulate HIV-1 production and/or viral replication. To test this, we generated TZM-bl cell lines that transiently express either HCV Env E1E2 or the soluble E2 (sE2) protein under drug selection (TZM-bl-E1E2 and TZM-bl-sE2, respectively). Plasmids were generated which expressed HCV E1E2 or sE2 carrying a V5 tagged epitope which allowed for testing transfected cell lines and confirming protein expression (**Supplementary Figure 1**). HIV-1 replication was monitored on TZM-bl, TZM-bl-E1E2 and TZM-bl-sE2 cell lines using replication competent LAI-YFP virus (1x10^3^ TCID_50_/ml input) and sampling viral p24 production (**Figure 1C**). Viral replication was highest on TZM-bl cells with a delay in viral replication observed on TZM-bl-sE2 cells, whereas replication was suppressed on TZM-bl-E1E2 cells (**Figure 1C**).

We next investigated the effects of HCV Env glycoprotein expression on suppressing HIV-1 activation in a cell line used as a model of HIV-1 viral latency (J-Lat-10.6). This cell carries a single integrated HIV-1 genome that can be activated with Tumour Necrosis Factor α (TNFα). An array of plasmid constructs (pCDNA, E1E2 and sE2) were transfected into J-Lat-10.6 cells, which were subsequently activated with TNFα and monitored for viral p24 production (**Figure 1D**). In the absence of TNFα activation, expression of viral p24 from J-Lat 10.6 was measured over several experiments and was consistently below the limit of ELISA detection (data not shown). Expression of both HCV E1E2 and sE2 suppressed induction of viral activation (P<0.05) in comparison to pCDNA.

### HCV Glycoproteins Repress HIV-1 TR Activity

**Figure 2:**
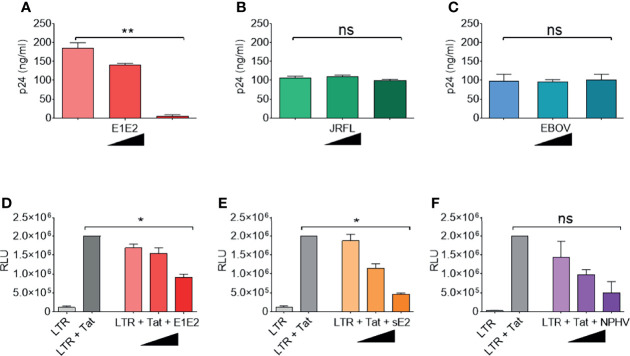
HCV E1E2 and sE2 Env proteins down-modulate HIV-1 LTR activity. **(A–C)** the p24 ng/ml quantification of virus generated by transfection of ΔEnv HIV-1 plasmid in conjunction with with increasing concentrations (9 ng, 90 ng and 900 ng) of the following: **(A)** HCV E1E2 Env expression plasmid (n = 4), **(B)** the HIV-1 JR-FL Env expression plasmid and (n = 4) **(C)** Ebola virus GP Env expression plasmid (n = 4). **(D–F)** LTR activation in 293T cells as quantified by luciferase when 6 ng LTR was co-transfected with HIV-1 1 ng Tat expression plasmid and in conjunction with different concentrations of **(D)** HCV E1E2 Env expression plasmid (n = 4) **(E)** HCV sE2 Env expression plasmid and (n = 4) **(F)** NPHV Env expression plasmid at three different concentrations for each (n = 4). Kruskal-Wallis and Dunn’s test were used to analyse significance. *P < 0.05, **P < 0.01 and ns – not significant. For all graphs, mean is plotted and error bars represent standard deviation and black triangles are used to depict increasing concentrations of plasmid (from 1 ng, to 6 ng and to 12 ng).

The above indicated that the HCV E1E2 Env protein has the capacity to reduce HIV-1 activity. We next tested the effects of increasing concentrations of different Env proteins on modulating viral production and demonstrated that higher concentrations of E1E2 transfection limited viral production (P<0.01) (**Figure 2A**), which was not observed for either HIV-1 Env (JR-FL) or Ebola virus GP (**Figures 2B, C**, respectively). In order to examine this HCV dependent effect on modulating LTR activity, different concentrations of E1E2 or sE2 were co-transfected along with a plasmid containing the luciferase (Luc) gene under the control of an HIV-1 LTR promotor (HIV-1 subtype B) into 293T cells (**Figures 2D, E**, respectively). Additionally, the HIV-1 Tat protein was co-transfected with the LTR plasmids to enhance LTR activation, which was quantified by measuring luciferase activity in transfected cell lysate. Inhibition was observed with both increasing concentrations of E1E2 (P<0.05) (**Figure 2D**) and sE2 (P<0.05) (**Figure 2E**), with sE2 showing a stronger inhibitory effect. In addition, we tested the homologous non-primate hepacivirus (NPHV) E1E2 protein for down-modulation of HIV-1 LTR activity and found a similar dose dependent decrease in LTR activation (P<0.05) (**Figure 2F**). Further, to ensure that the observed down-modulation was not due to toxicity of transfection or expression of the envelopes themselves, cell viability following transfection with various envelopes was measured (**Supplementary Figure 5D**). This indicated that viral Env proteins with sufficient homology to the HCV E1E2 protein can repress HIV-1 LTR activity.

**Figure 3:**
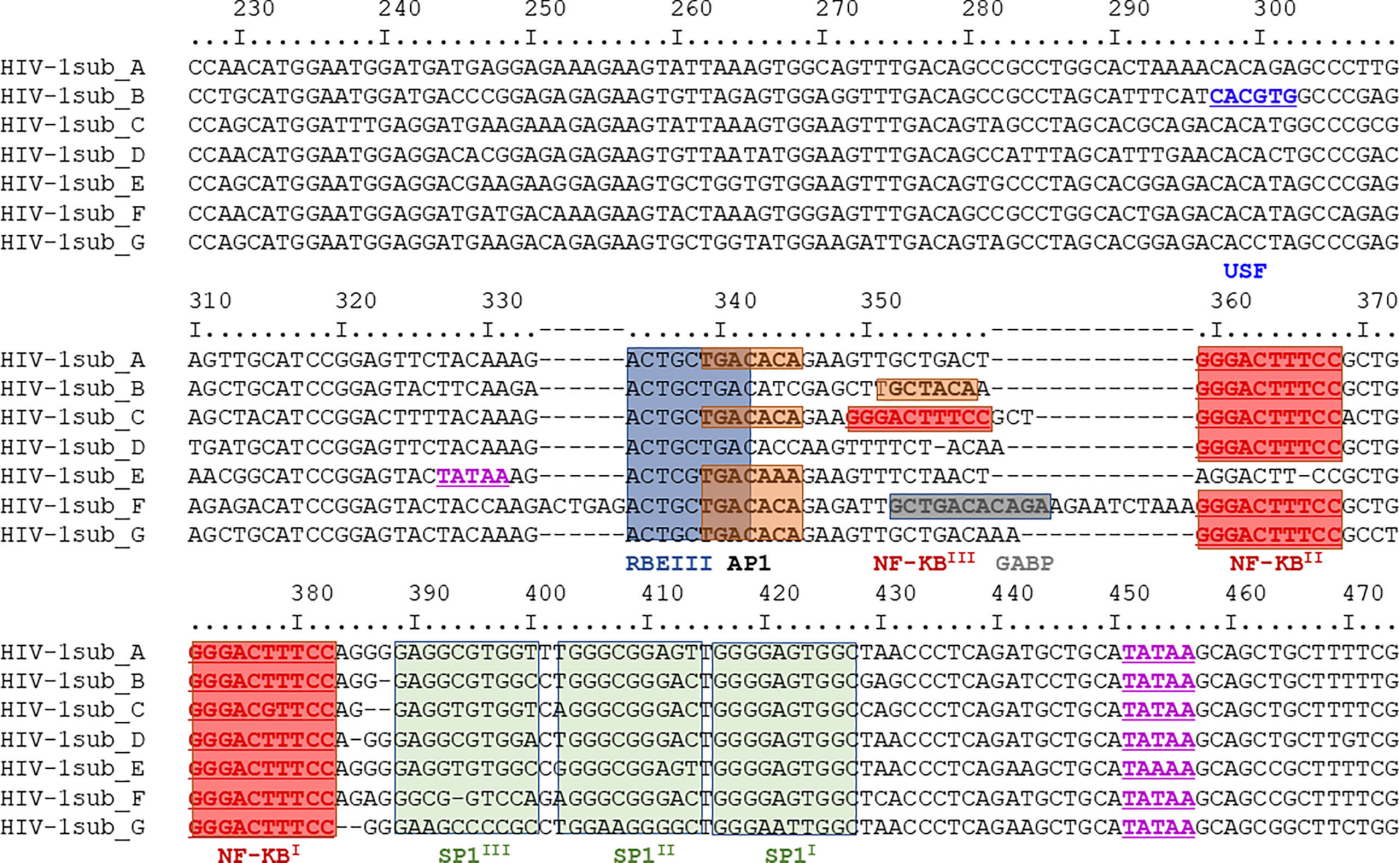
Nucleotide sequence comparison of HIV-1 LTR subtypes A-G. The region depict covers nucleotides 229 – 455 and with transcription factor binding sites highlighted: USF sites in dark blue, TATAA sites in purple, RBEIII sites in light blue, AP1 sites in orange, NF-κB sites in red, GABP sites in dark grey and SP1 sites in green. This same region represents the DNA subtype B LTR pull-down probe described in section 3.5.

**Figure 4:**
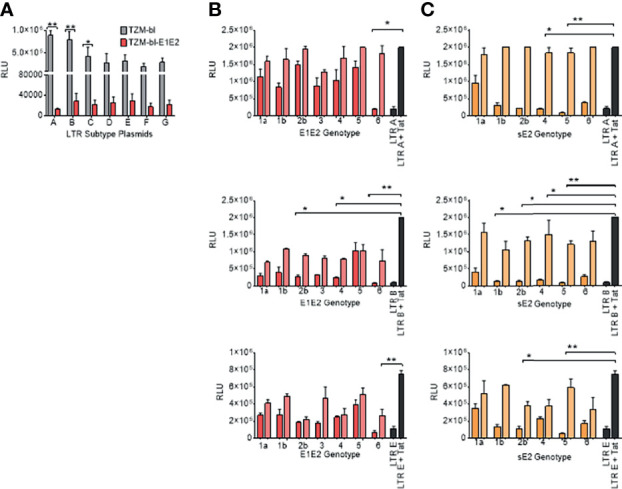
E1E2 and sE2 down modulates LTR activity of variant HIV-1 sub-types and by different HCV Env genotypes. **(A)** Transfection of LTR-luc plasmids into two cell lines: TZM-bl (grey) and TZM-bl-E1E2 (red) with 1 ng Tat plasmid also transfected in to activate the LTRs (n = 3). LTR activity was quantified *via* luciferase RLUs. **(B)** HIV-1 LTR-A, LTR-B and LTR-E activation when 293T cells co-transfected with two concentrations of E1E2 plasmid (12 ng represented by dark red bars, and 1 ng represented by light red bars) (n = 3) or **(C)** HIV-1 LTR-A, LTR-B and LTR-E activation when 293T cells co-transfected with two concentrations of sE2 plasmid (12 ng represented by dark orange bars, and 1 ng represented by light orange bars) (n = 3). For LTR control condition, 6 ng LTR-luc was transfected alone or in combination with 1 ng Tat expression plasmid. For all graphs, mean values are plotted and error bars represent standard deviation and statistical comparisons were performed utilising a Kruskal-Wallis and Dunn’s test comparing the activity between the control LTR + Tat transfection and with variant concentrations of E1E2 or sE2. *P < 0.05. **P < 0.01.

Due to the E1E2 and sE2 effects observed above, we aimed to determine whether E1E2 derived from different HCV genotypes could similarly affect the activity of multiple HIV-1 LTR subtypes. Differences are present in the number and type of transcription factor binding sites present within the HIV-1 LTR as shown (**Figure 3**). Therefore, to evaluate the effect of HCV Env E1E2 on HIV-1 LTR activity, a Tat expressing plasmid as well as a panel of plasmids containing HIV-1 variant subtype specific LTRs cloned upstream of a reporter Luc gene were transfected into TZM-bl and TZM-bl-E1E2 cells. LTR activity was quantified *via* luciferase activity and there was a generalised E1E2 mediated down-modulation of LTR activity across all HIV-1 subtype-specific LTRs tested (LTRs A, B, C, D, E, F and G) (**Figure 4A**). Additionally, to ensure that down-modulation of LTR activity was not due to toxicity of E1E2 expression or of transfection of LTR subtypes, viable cell counts were compared between TZM-bl and TZM-bl-E1E2 cells (**Supplementary Figure 5B**) as well as between 293T and 293T-E1E2 cells (**Supplementary Figure 5A**) transfected with various LTR subtypes.

We next sought to determine the effects of individual HCV E1E2 or sE2 Env genotypes (1a, 1b, 2b, 3, 4, 5, 6) against three selected HIV-1 LTR promoter subtypes (A, B and E). Two concentrations of E1E2 or sE2 plasmids (12ng and 1ng) were co-transfected along with the LTR plasmids in conjunction with the Tat expressing plasmid. Transfection of LTR subtype A with E1E2 demonstrates the ability of E1E2 to down-modulate LTR activation (**Figure 4B**), with the higher concentration showing more inhibition. When testing LTR subtype B, there is again a generalised down-modulation of LTR activity with the higher concentration of E1E2 Env plasmid (**Figure 4B**). With the subtype E LTR construct, a higher frequency of E1E2 genotypes did not demonstrate dose-dependent down-modulation of LTR expression (2b, 4 and 5) whereas other genotypes did and in general, lower expression was observed with sE2 for all genotypes (**Figure 4B**). When transfecting HIV-1 LTR A in conjunction with the sE2 expressing plasmid, a greater effect on LTR down-modulation was observed in comparison to E1E2 (**Figure 4C**). With regards to LTR B, the differences between LTR activation profiles with sE2 (**Figure 4C**) was shown to be greater than with E1E2 (**Figure 4C**). For example, there is a 6-fold difference in LTR activation between HCV Env genotype 4 with sE2 expression (12ng and 1ng) in comparison to a 3-fold difference with genotype 4 with E1E2. In addition, with genotype 5 and sE2 there was a 7-fold difference between 12 and 1ng in comparison to the smaller and relatively similar decrease with regards to genotype 5 and E1E2 Env. Similarly, when LTR E was transfected with sE2, as with LTR B, genotype 5 showed a greater effect than when transfected with E1E2 (**Figure 4C**). It should be noted that LTR E contains only one NF-κB binding site as opposed to the two found in subtypes A and B, and that the down-modulatory effect of E1E2/sE2 remains consistent (**Figures 4B, C**). Whilst statistical significance in relation to down-modulation of LTR activity is not observed across all conditions, the same trend is observed, indicating a generalised effect of E1E2 or sE2 on LTR activity irrespective of HCV genotype and where sE2 shows the greater down-modulation.

### HCV Glycoproteins Repress HIV-1 LTR Activity *via* an NF-κB Dependent Process

**Figure 5:**
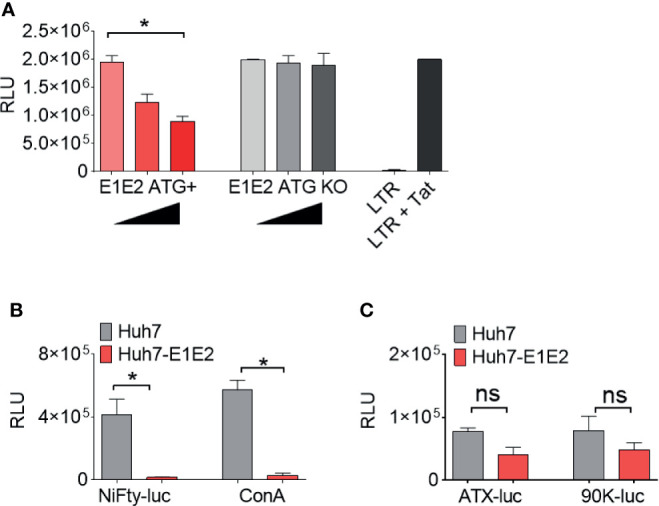
The down-modulation of LTR activity by HCV E1E2 is protein dependent and functions *via* NF-κB. **(A)** The total activation of HIV-1 LTR A when co-transfected with 3 concentrations of E1E2 envelope and an E1E2 KO mutant +/- on 293T cells (n = 4). Black triangles indicate an increase in plasmid concentration. The LTR was also transfected alone as a control for overall LTR activation. **(B, C)** Transfection of **(B)** NF-κB dependent and **(C)** non- NF-κB dependent promoters into Huh7 or Huh7-E1E2 stable cells (n = 4). LTR or promoter activation was quantified *via* luciferase (RLUs). For all graphs, mean values are plotted and error bars represent standard deviation and statistical comparisons were performed utilising Kruskal-Wallis and Dunn’s test to analyse significance. *P < 0.05. ns – non-significant.

To identify whether the effect on LTR activation is a result of E1E2 or sE2 protein and not an mRNA mediated effect, an HCV E1E2 genotype 6 knock-out (KO) mutant plasmid was generated and used to transfect 293T cells. It was observed that LTR activity was down-modulated in the presence of E1E2 protein (ATG+) (P<0.05) but not when E1E2 protein expression was knocked-out (ATG – KO Mut) (**Figure 5A**). Since the NF-κB pathway accounts for a significant proportion of LTR activation, this was chosen as a target for further investigation. To determine whether HCV glycoprotein mediated repression of HIV-1 LTR was most likely a result of affecting the NF-κB signalling pathway, a series of reporter plasmids were transfected into Huh7 cells or Huh7 cell lines stably expressing E1E2. Both NiFty-luc (5x NF-κB binding sites) and ConA (3x NF-κB binding sites) are plasmids that express luciferase under the control of NF-κB dependent promoters and show a significant decrease in luciferase activity in the presence of E1E2, by 28-fold (P<0.01) and 19-fold (P<0.01), respectively (**Figure 5B**). Conversely, ATX-luc and 90K-luc are plasmids that express luciferase under the control of general cellular promoters (autotaxin and 90K) and are not reliant on NF-κB and demonstrated no significant difference in luciferase activity in the presence of E1E2. Taken together, these results indicate that the previously observed E1E2 dependent reduction in LTR activity is a result of disruption to NF-κB signalling, which is protein (not RNA) dependent (**Figure 5C**).

### E1E2 Down-Modulation Associates with Disruption of Transcription Factors, Including NF-κB Binding to the HIV-1 LTR

The NF-κB signalling network is a diverse and complex series of pathways. As NF-κB dependent promoter activity was down-modulated by the presence of E1E2, we aimed to identify the mechanism by which this occurs. We analysed, in an unbiased fashion, the differential binding of proteins to the HIV-1 LTR in the absence or presence of the HCV E1E2 protein. The affinity purification followed by mass spectrometry (AP-MS) strategy utilises a double stranded LTR DNA capture probe to pull-down proteins from cells transfected with pCDNA control plasmid or with plasmid expressing HCV E1E2 (the natural Env protein being expressed in infected cells) and analysed the nuclear extract from these cells. The LTR probe was generated as a PCR template of WT HIV-1 5’LTR (subtype B, nucleotides 229 – 455) (covering the region as depict in **Figure 3**) before being incubated with nuclear extract generated from the two target cell lines (TZM-bl and TZM-bl-E1E2). The probe-protein complexes were treated with trypsin before analysis by reverse phase liquid chromatography and mass spectrometry to identify the probe-bound proteins (46). Gene ontology analysis with DAVID was performed on the 187 proteins confidently identified. The two most enriched processes were “Transcription” (64 proteins; p-value 5.1 10^-18^) and “Transcription Regulation” (53 proteins; p-value 9.4 10^-12^), based on normalized spectral count analysis of the data, a large number of proteins were found to differentiate in binding to the HIV-1 LTR, with 22 proteins binding in the presence of HCV E1E2 and 34 proteins enriched in the control experimental conditions (**Supplementary Table 1**). Detailed analysis of these 56 differentially bound proteins revealed that most of them possess DNA binding activity and/or transcription regulation properties, as indicated by the Uniprot database. In addition, all the proteins in italics in **Table 1** have been previously reported to play a role in HIV-1 replication (according to PubMed), consistent with the high quality and relevance of this data.

**Table 1:** Effect of the presence of E1E2 on the proteins captured by the HIV-1-LTR.

Protein name	Accession number	DNA binding	Transcript. regul.	E1E2	Control
**Significantly enriched in control samples**					
*Nuclear factor NF-kappa-B p105 subunit*	NFKB1_HUMAN	Y	Y	1	14
*Nuclear factor NF-kappa-B p100 subunit*	NFKB2_HUMAN	Y	Y	0	41
*RELA protein*	Q2TAM5_HUMAN	Y	Y	0	6
*Cluster of Nuclear receptor subfamily 2 group C member 2*	NR2C2_HUMAN	Y	Y	0	4
*Cluster of COUP transcription factor 1*	COT1_HUMAN	Y	Y	2	43
*Cluster of X-ray repair cross-complementing protein 5*	XRCC5_HUMAN	Y	Y	34	76
*X-ray repair cross-complementing protein 6*	XRCC6_HUMAN	Y	Y	61	118
*Upstream stimulatory factor 1*	USF1_HUMAN	Y	Y	0	7
*Upstream stimulatory factor 2 (Fragment)*	M0QXT0_HUMAN	Y	Y	0	10
*Transcription factor Sp1*	SP1_HUMAN	Y	Y	14	34
*Cluster of Gamma-interferon-inducible protein 16*	IFI16_HUMAN	Y	Y	0	132
*Paired amphipathic helix protein Sin3a*	SIN3A_HUMAN	/	Y	0	16
*Cluster of Leucine-rich PPR-motif containing*	E5KNY5_HUMAN	Y	/	0	5
*Three prime repair exonuclease 1*	Q5TZT0_HUMAN	Y	/	0	8
Cluster of Histone H2B type 1-C/E/F/G/I	H2B1C_HUMAN	Y	/	0	7
Cluster of Steroid hormone receptor ERR1	ERR1_HUMAN	Y	Y	0	8
DNA ligase 3	DNLI3_HUMAN	Y	/	0	22
Cluster of Histone H3	B2R4P9_HUMAN	Y	/	2	6
Cluster of Nuclear factor 1 A-type	NFIA_HUMAN	Y	Y	0	9
Cluster of Elongation factor 1-alpha 1	EF1A1_HUMAN	Y	/	2	18
DNA topoisomerase 2-alpha	TOP2A_HUMAN	Y	/	39	225
Cluster of DNA topoisomerase 2-beta	TOP2B_HUMAN	Y	/	22	265
Circadian locomoter output cycles protein kaput	CLOCK_HUMAN	Y	Y	0	11
Zinc finger BED domain-containing protein 6	ZBED6_HUMAN	Y	Y	0	13
Nuclear receptor V-erbA-related	F1D8R3_HUMAN	Y	Y	0	11
Cluster of Class E basic helix-loop-helix protein 40	BHE40_HUMAN	Y	Y	0	9
Cluster of DNA topoisomerase 1	TOP1_HUMAN	Y	/	8	55
Krueppel-like factor 13	KLF13_HUMAN	Y	Y	4	10
Cluster of Homeobox protein TGIF1	TGIF1_HUMAN	Y	Y	0	5
Mitochondrial DNA polymerase subunit gamma-2	E5KS22_HUMAN	Y	/	25	43
Endoplasmic reticulum chaperone BiP	BIP_HUMAN	/	/	0	13
Cluster of Epididymis secretory sperm binding protein Li 124m	V9HW84_HUMAN	/	/	1	10
p180/ribosome receptor	A7BI36_HUMAN	/	/	5	25
Cluster of Heat shock cognate 71 kDa protein	E9PKE3_HUMAN	/	/	9	29
					
**Significantly enriched in E1E2 samples**					
*Helicase-like transcription factor*	HLTF_HUMAN	Y	Y	54	4
*Cluster of RNA-binding motif protein, X chromosome*	RBMX_HUMAN	Y	Y	22	0
*Cluster of Non-POU domain-containing octamer-binding protein*	NONO_HUMAN	Y	Y	29	2
*Poly [ADP-ribose] polymerase 1*	PARP1_HUMAN	/	Y	768	388
*Nucleolin*	NUCL_HUMAN	Y	Y	19	2
Cluster of Lamina-associated polypeptide 2, isoform alpha	LAP2A_HUMAN	Y	Y	9	0
Cluster of cDNA FLJ54552, highly similar to Heterogeneous nuclear ribonucleoprotein K	B4DUQ1_HUMAN	Y	Y	86	33
Leucine-rich repeat and WD repeat-containing prot. 1	LRWD1_HUMAN	Y	/	4	0
Telomere-associated protein RIF1	RIF1_HUMAN	/	Y	20	1
Cluster of Heterogeneous nuclear ribonucleoprotein M	HNRPM_HUMAN	/	/	9	0
Acyl-CoA dehydrogenase family member 11	ACD11_HUMAN	/	/	26	0
Cytoskeleton-associated protein 5	CKAP5_HUMAN	/	/	28	0
Cluster of Glutamine--tRNA ligase	SYQ_HUMAN	/	/	12	0
Cluster of Epididymis luminal protein 189	Q5HYB6_HUMAN	/	/	31	1
cDNA, FLJ96156, highly similar to Homo sapiens leucyl-tRNA synthetase (LARS), mRNA	B2RCM2_HUMAN	/	/	14	0
Cluster of cDNA FLJ52761, highly similar to Actin, aortic smooth muscle	ACTBL2_HUMAN	/	/	279	32
Methionine--tRNA ligase, cytoplasmic	SYMC_HUMAN	/	/	5	0
Cluster of Myosin light polypeptide 6	MYL6B_HUMAN	/	/	42	0
Cluster of Myosin-10	MYH10_HUMAN	/	/	482	0
Cluster of Kelch-like protein 7	KLHL7_HUMAN	/	/	14	8
Myosin regulatory light chain MRCL3 variant (Fragment)	Q53HL1_HUMAN	/	/	23	0
Bifunctional glutamate/proline--tRNA ligase	SYEP_HUMAN	/	/	24	0

Nuclear extracts proteins from TZM-bl cells transiently transfected with E1E2 plasmid (“E1E2”) or with pCDNA plasmid (“control”) were pulled-down with a HIV-1-LTR DNA fragment and identified by mass spectrometry. A quantitative analysis was performed to compare the normalized spectral count in E1E2 and control conditions, with a T-test comparison. Proteins with significant differences (p-value < 0.05) are shown, while all the results are presented in **Supplementary Table 1**. E1E2 S.C. and Control S.C. represent the merge of the normalized spectral counts of 3 biological independent replicates of the corresponding experimental condition. The mention of DNA-Binding and of Transcription Regulation activities was manually search in Uniprot database, and indicated by Y when present. The proteins reported to have a link with HIV (PubMed search) are indicated in italics. E1E2 S.C. and Control S.C. represent the merge of the normalized spectral counts of each replicate of the corresponding experimental condition.

When comparing the proteins binding to HIV-1 LTR some clear differences were found (**Table 1**). The most interesting findings being that several positive transcriptional regulators of HIV1-LTR transcription were found exclusively or more abundantly in the HIV-1 LTR pull-down performed in the absence of E1E2, suggesting that these positive regulators are inhibited by E1E2 expression. This concerns 3 members of the NF-κB family (NF-κB1, NF-κB2 and RelA), the Specificity Protein 1 (SP1), which was one of the very first positive regulator of HIV-1 transcription described (56), COUP TF, which stimulates HIV-1 transcription (57), in cooperation with Nuclear receptor subfamily 2 group C member 2 (NR2C2), also called thyroid receptor 4 orphan receptor (58). The same differential pull-down profile is observed for the DNA repair proteins XCCR5 and XCCR6 compose the Ku complex, described to bind directly to the HIV-1 LTR, promoting early transcription from the promoter (59). Unexpectedly, while the interferon gamma inducible protein 16 (IFI16) was exclusively found in the HIV-1 LTR pull-down in the absence of E1E2, this protein is described to target the SP1 positive transcription factor to suppress HIV-1 transcription (60). Amongst the proteins that bound to the LTR probe in the presence of E1E2, we found the non-POU domain-containing octamer-binding protein (NoNO), a transcription factor that negatively regulates HIV-1 infection in T lymphocytes (61), Helicase-like transcription factor (HLTF), that has been shown to restrict HIV-1 replication (62), PARP-1, a negative regulator of HIV-1 transcription (63) and replication (64) or RBMX which has recently been shown to bind the HIV-1 LTR proviral DNA to maintain the viral latency (65).

The differential binding profile of transcriptional regulators identified in the DNA pull-down is consistent with the data obtained with the HIV-1 LTR luciferase reporter and HIV-1 replication, since most negative regulators are found in the experimental condition where E1E2 is expressed, while the positive regulators are more abundant in the absence of E1E2. However, the mechanisms by which E1E2 induces this switch is currently not known. The results suggest a wide-range of alterations in transcription factors binding to the HIV-1 LTR associated with HCV E1E2 Env expression. We selected a number of the transcription factors associated with our findings (NF-κB p50, RelA, IFI16 and RBMX) to identify whether the protein binding alterations observed correlated to alterations at the mRNA expression levels. Quantification assays were developed for each gene, but no differences were found in mRNA expression for NF-κB, RelA, IFI16 or RBMX (**Supplementary Figures 2A–D**). These results demonstrate a scenario where the disruption to NF-κB binding the LTR is *via* post-transcriptional mechanism.

### HCV Glycoproteins Activate Host Endoplasmic Reticulum Stress Pathways Known to Inhibit NF-κB Activity

**Figure 6:**
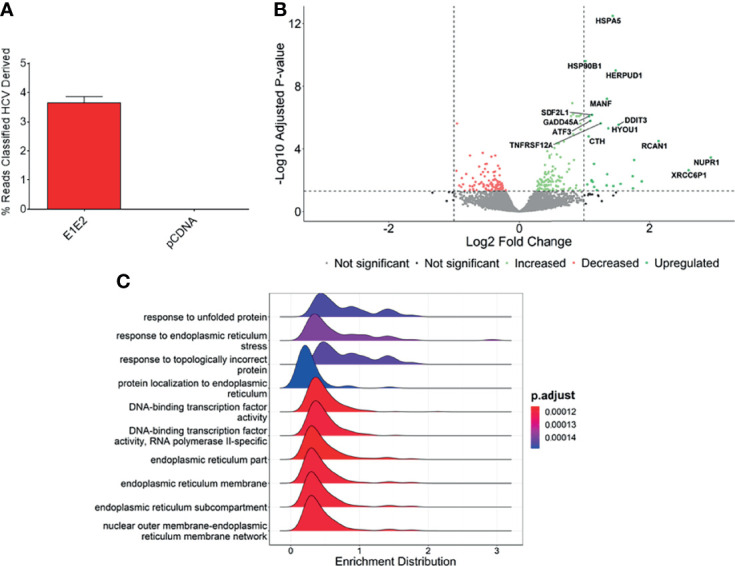
Genes associated with endoplasmic reticulum stress are upregulated in the presence of E1E2. **(A)** Percentage of reads mapped to HCV genome in pCDNA (n = 4) or E1E2 (n = 6) transfected 293T cells, using Kraken2. **(B)** Volcano plot highlighting the significantly upregulated genes, based on Log2 fold change >1 or -Log10 adjusted P-value above 1.3 (P=0.05). **(C)** Ridgeplot showing top 10 biological processes that are enriched in our dataset. Differentially expressed genes are involved in misfolded protein binding and endoplasmic reticulum stress.

**Figure 7:**
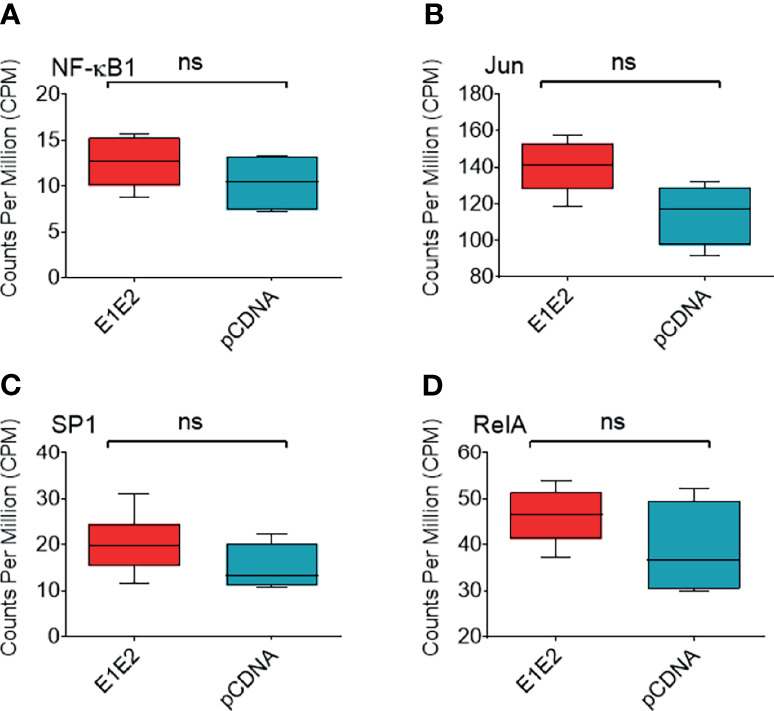
Expression of NF-κB associated or HIV-1 transcription factor genes. Expression of NF-κB associated or HIV-1 transcription factor genes from normalised RNAseq libraries of 293T cells expressing E1E2 (n = 6) or pCDNA (n = 4), expressed as counts per million (CPM) **(A)** NF-κB1, **(B)** Jun, **(C)** SP1 and **(D)** RelA. Significance determined by Voom/Limma differential gene expression analysis. ns - not significant.

We next measured differential gene expression (DGE) patterns between cells expressing the HCV E1E2 Env protein in comparison to cells transfected with the control vector, pCDNA (GEO accession: GSE163239). We selected to utilise the 293T cell line for differential gene expression analysis to provide consistency with results showing the effect of E1E2 expression on down-modulating HIV-1 LTR activity (section 3.2). Since the down-modulation of HIV-1 LTR activity in the presence of HCV E1E2 Env protein is similar between 293T and Huh7 cells we are confident our results are reflective of cell types infected with HCV. Initially, to demonstrate expression of E1E2, all the expression libraries were mapped to an HCV reference genome, highlighting expression of HCV derived genes only in the E1E2 transfected cells (**Figure 6A**). The effect of HCV glycoproteins on the cellular transcriptome was analysed using the Limma/Voom package following assessment of library length and quality distributions and sample clustering *via* principal component analysis (PCA) (**Supplementary Figures 3A-C**). Additionally, libraries were transformed and normalised using EdgeR, according to the method described (50–52) (**Supplementary Figures 3D, E**). Several genes were significantly upregulated in the presence of E1E2 when compared to control pCDNA transfected cells, and no genes were significantly downregulated (**Figure 6B**). In line with our previous experiments, we sought to determine the effect of E1E2 on the expression of cellular transcription factors such as NF-κB, that are known to interact with the HIV-1 LTR. In our dataset, expression of NF-κB, SP1, RelA and Jun were not significantly affected by the presence of E1E2 (**Figures 7A–D**).

The most significantly enriched biological processes are highlighted (**Figure 6C**), which shows that pathways involved in the endoplasmic reticulum (ER) stress response are enriched in the presence of E1E2 (**Figure 6C** and **Supplementary Figure 4**). Further, our analysis showed that the most significantly enriched pathways are all associated with ER stress or the cellular response to stress (**Supplementary Table 2** and **Supplementary Figures 4A–E**).

**Figure 8:**
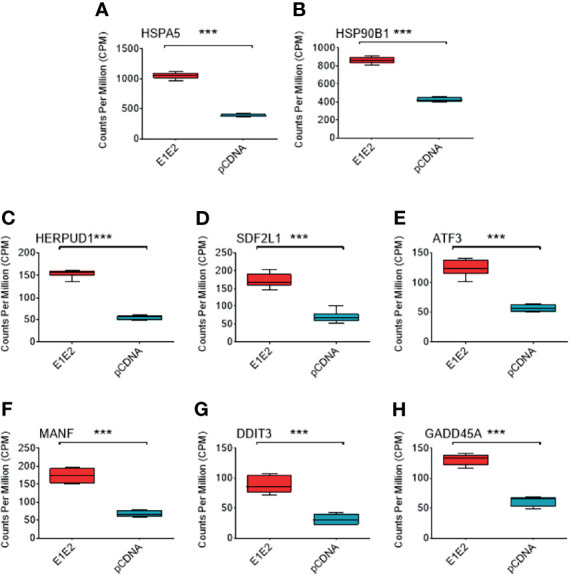
Comparison of different ER stress associated genes in DGE dataset between pCDNA (n = 3) and E1E2 (n = 3) transfected cells. **(A)** Comparison of HSPA5 expression. **(B)** Comparison of HSP90B1 expression. **(C)** Comparison of HERPUD1 expression. **(D)** Comparison of SDF2L1. **(E)** Comparison of ATF3 expression. **(F)** Comparison of MANF expression. **(G)** Comparison of DDIT3 expression. **(H)** Comparison of GADD45A expression. Significance determined by Voom/Limma differential gene expression analysis and ns, not significant, *P < 0.05, **P < 0.01, ***P < 0.001.

Individual genes that are significantly upregulated in our data set are associated with ER stress. Heat shock protein family A (Hsp70) member 5 (HSPA5) gene encodes the binding immunoglobulin protein (BiP) and is the most significantly upregulated gene in our dataset (**Figures 6B**, **8A**). BiP is the master regulator of the UPR, and as such its overexpression indicates a cellular response to ER stress and explains the upregulation of other genes such as heat shock protein 90 beta family member 1 (HSP90B1) (**Figure 8B**), homocysteine inducible ER protein with ubiquitin like domain 1 (HERPUD1) (**Figure 8C**), stromal cell derived factor 2 like 1 (SDF2L1) (**Figure 8D**), activating transcription factor 3 (ATF3) (**Figure 8E**), mesencephalic astrocyte derived neurotrophic factor (MANF) (**Figure 8F**), DNA damage inducible transcript 3 (DDIT3) (**Figure 8G**) and growth arrest and DNA damage inducible alpha (GADD45A) (**Figure 8H**). The upregulation of these genes strongly suggests that the UPR is upregulated in the presence of HCV E1E2 Env expression.

### Knock-Down of ATF3 Expression Alleviates the Inhibitory Effects of E1E2 HCV Env on HIV-1 LTR Activity

Based on the above finding that ATF3 (a major component to the ER stress pathway) was significantly up-regulated in the presence of E1E2 we aimed to identify whether knocking-down the expression of this specific gene could modulate the E1E2 inhibitory effect observed. This is also linked with previous reports that ATF3 negatively regulates NF-κB activity (66, 67), To this end, 293T cells were transfected with three independent shRNA constructs targeting ATF3 in comparison to either a pCDNA control or ATF3 scrambled shRNA plasmid. Initially, to ensure that transfection of the shRNA constructs was not toxic to the cells, viability following transfection was measured (**Supplementary Figure 5C**). Inhibition of ATF3 production was demonstrated by Western blot analysis, which revealed reduced ATF3 expression in the presence of the shRNA constructs but not in the presence of control constructs such as scrambled shRNA and pCDNA (**Supplementary Figure 6A**).

To identify whether knocking-down ATF3 could modulate NF-κB dependent responses we transfected the above generated ATF3 knock-down cells with HIV-1 LTR-Luc (NF-κB dependent) and Tat expression plasmids and measured Luc activity 48 h later. Pre-transfection with the three ATF3 directed shRNA plasmids provided a higher level of Luc activity than with control plasmid alone (P<0.05) (**Supplementary Figure 6B**). To assess the effect of E1E2 expression cells were similarly pre-transfected independently with the three ATF3 shRNA, or scrambled shRNA plasmids, before being transfected with E1E2, HIV-1 LTR-Luc and Tat expression plasmids and with Luc activity subsequently measured. When cells were pre-transfected with the scrambled shRNA then the typical profile of E1E2 down-modulation of LTR expression was observed (**Supplementary Figures 6C–E**, shown in red). Whereas, Knock-down of ATF3 expression was shown to negate the HCV E1E2 inhibitory effect on HIV-1 LTR activity (P<0.05) when pre-transfecting with ATF3 shRNA 1 (**Supplementary Figure 6C**, shown in green), ATF3 shRNA 2 (**Supplementary Figure 6D**, shown in green) and ATF3 shRNA 3 plasmids (**Supplementary Figure 6E**, shown in green). This result demonstrates that knocking-down ATF3 expression, one of the major components of the ER stress pathway, alleviates the inhibitory effect of E1E2 on down-modulating HIV-1 LTR activity and induced NF-κB responses. The results additionally suggest that basal expression levels of ATF3 can influence HIV-1 LTR expression activity independent of E1E2 expression.

## Discussion

The work presented here demonstrates that the HCV E1E2 Env glycoproteins can modulate HIV-1 LTR through downregulation of NF-κB mediated immune signalling pathways. The findings presented here provides a proof-of-concept where a novel pathway exists whereby a viral Env protein (HCV) can modulate NF-κB signalling, potentially leading to pronounced effects on an array of biological phenomena.

We have demonstrated an increased effect by sE2 alone to down-modulate HIV-1 LTR activity. Soluble E2 (sE2) has been used previously as a means to map antibody epitopes (68), CD81-related entry mechanisms (69) and DC/L-SIGN-mediated receptor binding (70). Whilst it is unclear whether sE2 is produced as a soluble glycoprotein during infection, E2 can be found in the cytoplasm of infected cells whereby it can modulate intracellular signalling pathways (expanded upon below).

### The Role of E1E2 or sE2 as a Transcription Regulatory Factor

HCV E2 has previously been documented to be capable of modulating several intracellular signalling pathways, including the MAPK/ERK (71), PI3/AKT (72) and PKR/eIF2 pathways which lead to an increase in cell proliferation and survival as well as a means to enhance viral infectivity. E2 has also been shown to inhibit other signalling pathways including T-cell signalling in a cross-genotypic RNA dependant manner by reducing Lck phosphorylation (73). This in turn was shown to prevent downstream TCR signalling. Since the PKR, Lck and PI3K-AKT pathways can indirectly influence NF-κB signalling (74) this suggests a global means by which sE2 can influence cellular transcription. Similarly, here we demonstrate that sE2, either alone or as part of the E1E2 heterodimer, may also modulate the NF-κB pathway, either directly or indirectly through stimulation of inhibitory pathways.

### A Potential Mechanism for HCV Derived Modulation of NF-κB Activity

Three members of the NF-κB family were detected exclusively [RelA and NF-κB2 (p100, the precursor form of p52)] or more abundantly [NF-κB1 (p105, precursor form of p50)] in the HIV-1-LTR pull-down performed in the absence of E1E2. This suggests that, although the expression (**Figure 7D** and **Supplementary Figure 2B**) of RelA is comparable in the presence or absence of E1E2, E1E2 inhibits NF-κB binding specifically to the HIV-1 LTR. Although speculative, one can hypothesize that E1E2 inhibits a RelA-targeting kinase, such as ERK, thereby modifying the DNA-binding affinity of NF-κB specifically for the kB sequence present in the LTR. Indeed, post-translation modifications of NF-κB subunits determine the sequence-specific binding of this transcription factor (75). Alternatively, the absence of HIV1-LTR-bound NF-κB might be due to the absence of the other protein partners detected in the pull-down in the absence of E1E2.

### Link Between HCV and Endoplasmic Reticulum Stress

Expression of HCV proteins or replication of HCV in tissue culture models is associated with ER stress and subsequent activation of the UPR and inflammatory pathways due to the dependence on the ER for viral replication (35, 42, 76, 77). Several studies have suggested that activation of the UPR in HCV infected cells contributes to pathogenesis, including through increased liver fibrosis *via* transforming growth factor β1 (TGF-β1) induction (78) and development of insulin resistance and type 2 diabetes mellitus (79, 80). In line with these findings, we have shown that ER stress response pathways are significantly upregulated in the presence of E1E2. This supports studies which indicate E1E2 Env expression is a potent activator of ER stress (41, 42). Follow on experiments analysing more specifically the promoter regions of the ER stress dysregulated mRNA genes in the presence of E1E2 or the factors binding to these specific promoters may provide an indication as to whether altered expression of mRNA is due to E1E2 binding or the binding of other dysregulated TF induced by E1E2 expression.

The UPR is a signalling cascade that is activated when misfolded proteins accumulate in the ER and is detected by three molecular sensors: IRE1 (inositol-requiring 1α), PERK (double-stranded RNA-dependent protein kinase (PKR)-like ER kinase) and ATF6 (activating transcription factor 6) (34, 35). The activation of the UPR ultimately leads to restoration of cellular homeostasis by increasing the folding capacity of the ER and reducing protein synthesis by the upregulation of UPR effector proteins such as CHOP (DDIT3), BiP (HSPA5) and X-box binding protein 1 (XBP1) (81).

There is significant and complex interaction between the UPR and the cellular transcription machinery and evidence suggests that the UPR may cause both positive and negative regulation of NF-κB, depending on the specific conditions and the timing of the response, as reviewed (82, 83). We provide evidence that E1E2-mediated ER stress may lead to a reduction in NF-κB-dependent HIV-LTR activation, though expression of NF-κB genes were not differentially expressed in our analysis. This E1E2-associated down-modulation may therefore be *via* direct or indirect interaction with UPR effector proteins. One potential regulator is ATF3, a transcription factor that is involved in the host response to inflammation, infection and cancer (84–87). Several reports propose that ATF3 is a negative regulator of NF-κB mediated pro-inflammatory responses (88–90), including a study which showed elevated levels of immune activation through NF-κB signalling in *Drosophila melanogaster* ATF3 knockout mutants (91). A recent study indicates that ATF3 is a hub of transcriptional activity in HCV infected cells, suggesting a key role for this transcription factor in HCV infection and the response to cell stress (92). Importantly, research has shown that ATF3 directly binds the p65 NF-κB subunit, and that inhibition of ATF3 was associated with increased NF-κB activity (66). Taken together, these studies may indicate a mechanism by which HCV E1E2 activates the UPR, leading to overexpression of ATF3, a negative regulator of NF-κB. Indeed, we showed, through short hairpin RNAs targeting ATF3 transcripts, that inhibition of ATF3 expression was able to both enhance basal activation of the LTR as well as alleviate the effect of E1E2. These results suggest that E1E2 expression activates pathways that disrupt NF-κB signalling, specifically the stress response gene ATF3, and that this effect may be important in HCV mediated immune regulation. The precise mechanism *via* which the down-modulation of ATF3 leads to the down-modulation of NF-κB activity needs to be further elucidated but an association with ER stress has been demomstrated.

As the abundance of precursor proteins p100 and p105 were lower in the E1E2 containing sample, it would suggest that E1E2 modulates the production of these proteins. This may include p100 and p105 transcription/translation, or the phosphorylation process by which each protein is released from its inhibitory state. Since E2 is a documented modulator of intracellular signalling (as described above), it is proposed that E2 may be the means by which HCV modulates NF-ĸB signalling (as part of the E1E2 heterodimer complex) and future *in vitro* systems should clarify this.

### Impact on Latency and Other Biological Systems

Modulation of NF-κB signalling also has implications with regards to HIV-1 latency. The process of maintaining HIV-1 latency can be described as a balance between enabling infected cell survival and proliferation but preventing HIV-1 gene re-expression. To this end, HIV-1 employs multiple methods by which it maintains homeostatic cell proliferation and basal levels of transcription. With regards to NF-κB involvement these include chromatin accessibility, wherein transcription factors have access to proviral DNA to facilitate binding (93). It has been postulated that the level of chromatin accessibility can determine the required level of NF-κB binding for LTR activation (94). A number of molecules with known capacities to modulate HIV-1 latency were shown to be altered in LTR pull-downs in the absence of E1E2 expression (XCRR5 and 6) and which have been shown to enhance early expression from the HIV-1 LTR promoter (59). Pro-inflammatory cytokines (typically activated by NF-κB) also serve to replenish the HIV-1 reservoir by reactivating viral replication, however when this response is suppressed this helps maintain a homeostatic proliferation of the cellular reservoirs (95). Additionally, NF-κB (and NFAT) acts to reverse the epigenetic inhibition related to the LTR leading to proviral transcription *via* chromatin remodelling thus abolishing the histone deacetylase and methyltransferase related regulation (96). A recent study has described a higher HIV-1 proviral reservoir being identified in individuals treated for HIV-1 who were either co-infected with HCV or who resolved their HCV infection in comparison to mono-HIV-1 infected individuals (97). This could represent a mechanism whereby HCV Env expression can lead to down-modulation of HIV-1 LTR activity and induction of viral latency, not necessarily through co-infection of the same cell but *via* other mechanisms such as cellular uptake or cell fusion. There is evidence that HIV-1 can infect cells within the liver, namely primary hepatic stellate cells (HSC) where HCV replication occurs in adjacent hepatocytes. Indeed *in vitro* culture systems have indicated that liver fibrosis induction can be enhanced in HIV-1 infected individuals through complex interactions of cell-types infected with either HIV-1 or HCV (98, 99).

The potential wide-ranging modulation of various intra-cellular signalling pathways by sE2 suggests that in addition to affecting viral activity this mechanism has the potential to modulate an array of host cell phenotypes. This would undoubtedly include regulation of immune responses or cell proliferation leading to oncogenesis. NF-κB is required as part of a pro-inflammatory response and as such sustained activation of the NF-κB pathway leads to chronic inflammation and in some cases inflammation-associated cancer such as hepatocellular carcinoma (in the case of HCV-related disease) (100). Additionally, NF-κB acts to prevent apoptosis and thus prolong cell survival leading to tumour formation as well as sustaining angiogenic and metastatic factors such as vascular-endothelial growth factor and Twist1 (101). Therefore, the effect that E2 has on NF-κB or additional intracellular pathways needs to be carefully elucidated.

Overall, it is shown that HCV E2 possesses a wide-ranging capability to influence intracellular signalling events. The research presented here also demonstrates a novel consequence of HCV/HIV-1 co-infection in which E2 down-modulates LTR activity. Given that co-infection of the same cell is rare the major consequences to these findings will lie in the effect of the HCV E2 Env protein in majorly down-modulating NF-κB mediated pro-inflammatory as well as oncogenesis pathways in HCV infected individuals.

## Data Availability Statement

Data has been deposited in NCBI’s Gene Expression Omnibus and accessible through GEO Series accession number GSE163239 (https://www.ncbi.nlm.nih.gov/geo/query/acc.cgi?acc=GSE163239). All reagents generated will be available from the Lead Contact with a completed Materials Transfer Agreement.

## Author Contributions

GP and WP designed and perceived the study. GP, WP, JM, JB, AT and PR provided scientific input and direction as well as performing data analysis. LM, JT, AF, MD, WA and AR designed and performed experiments and undertook data analysis. JM, JB and AT provided reagents. LM and WP wrote the initial manuscript. All authors contributed to the article and approved the submitted version.

## Funding

The project was funded through the European Community’s Seventh Framework Programme under grant agreement nr. HEALTH-F3-2012-305578 (PathCo).

## Conflict of Interest

The authors declare that the research was conducted in the absence of any commercial or financial relationships that could be construed as a potential conflict of interest.

## Publisher’s Note

All claims expressed in this article are solely those of the authors and do not necessarily represent those of their affiliated organizations, or those of the publisher, the editors and the reviewers. Any product that may be evaluated in this article, or claim that may be made by its manufacturer, is not guaranteed or endorsed by the publisher.

## Supplementary Material

Supplementary Figure 1 FACS analysis of **(A)** TZM-bl cells, **(B)** TZM-bl cells and isotype control, **(C)** TZM-bl-E1E2 and **(D)** TZM-bl-sE2 stably expressing cells. The primary antibody used was AP33, a mouse monoclonal and the secondary was an anti-mouse Goat PE conjugate.

Supplementary Figure 2 E1E2 expression modulates an array of host protein transcription factors binding the HIV-1 LTR but not modulating mRNA expression Quantitative mRNA expression of transcription factors that bind the HIV-1 LTR in the presence or absence of HCV E1E2 Env expression (n = 3). **(A)** NF-κB **(B)** RelA **(C)** IF116 and **(D)** RBMX. All graphs show mean values plotted with error bars representing standard deviation.

Supplementary Figure 3 Overview of normalisation and quality control of differential gene expression data set including pCDNA (n = 4) and E1E2 (n = 6) transfected cells. **(A)** Distribution of read lengths in mRNA libraries. **(B)** Distribution of read qualities in mRNA libraries. **(C)** Principal component analysis highlighting separation of sample groups on principle component 1. **(D)** Distribution of gene expression (Log-CPM) prior to filtering with EdgeR. **(E)** Distribution of gene expression post-filtering with EdgeR.

Supplementary Figure 4 Gene set enrichment analysis showing the enrichment plots and corresponding heat maps of enriched gene sets that are associated with ER stress. **(A)** SRP dependent co-translational protein targeting to membrane. **(B)** PERK regulates gene expression. **(C)** Unfolded protein response (UPR). **(D)** ATF4 activates genes in response to ER stress. **(E)** IRE1ALPHA activates chaperones.

Supplementary Figure 5 Effect of transfection and protein expression on cell viability. **(A)** Comparison of viable cell counts in cells 293T cells and 293T-E1E2 cells that were transfected with LTR sub-type plasmids and expression constructs. **(B)** Comparison of viable cell counts in cells TZM-bl cells and TZM-bl-E1E2 cells that were transfected with LTR sub-type plasmids and expression constructs. **(C)** Comparison of cell viability when cells were transfected with ATF3 knock-down constructs including scrambled shRNA and the three ATF3 shRNA plasmids. TR represents transfection reagent, in which the transfection process was performed in the absence of plasmid DNA. **(D)** Comparison of cell viability when cells were transfected with various envelope expression plasmids. TR represents transfection reagent, in which the transfection process was performed in the absence of plasmid DNA.

Supplementary Figure 6 Knock-down of ATF3 expression through siRNA alleviates the inhibitory effects of E1E2 HCV Env on HIV-1 LTR activity. **(A)** Western blot showing the expression level of ATF3 in 293T cells transfected with ATF3 shRNA expression plasmids, HCV E1E2 Env and scrambled siRNA pCDNA controls (top panel) and β-actin loading control (bottom panel). **(B)** Total activation of HIV-1 LTR in 293T cells when cells were prior transfected with ATF3 shRNA expression plasmids (green bars) or pCDNA (grey bar) and scrambled shRNA (blue bar) controls (n = 3). Cells were initially transfected with siRNAs or pCDNA expression plasmid and 48h later were transfected with 50 ng LTR-Luc and 5 ng HIV-1 Tat plasmids and with Luc activity measured 48 h later from cell lysate. Letter codes used for transfection are as follows: L: LTR-luc, T: Tat, E: E1E2, P: pCDNA, A: ATF3 shRNA, S: scrambled shRNA. **(C–E)** demonstrate Luc activity from 293T cell lysates when cells were prior co-transfected with ATF3 shRNA expression plasmid and 48h later with E1E2 HCV Env, LTR-Luc and HIV-1 Tat plasmids and Luc activity measured 48h subsequently (green bars) (n = 3). Cells non-transfected with siRNA prior to E1E2 Env, LTR-Luc and HIV-1 Tat plasmid transfection were used as a positive control for monitoring the effects of E1E2 expression (red bars) and cells transfected with control LTR-Luc and HIV-1 Tat to determine basal Luc expression (grey bars). The inhibitory effects of E1E2 HCV Env on HIV-1 LTR activity is alleviated in the presence of **(C)** ATF3 shRNA 1, **(D)** ATF3 shRNA 2 and **(E)** ATF3 shRNA 3 when compared with controls. The same letter codes described above for panel B were also used for panels **(C–E)**. For all graphs, mean is plotted and error bars represent standard deviation. Kruskal-Wallis and Dunn’s test were used to determine statistical significance. * P<0.05.

Supplementary Table 1 Identification of the proteins captured by the HIV-1-LTR in the presence or absence of E1E2. Nuclear extracts proteins from TZM-bl cells transiently transfected with E1E2 plasmid (“E1E2”) or with pCDNA plasmid (“control”) were pulled-down with a HIV-1-LTR DNA fragment and identified by mass spectrometry. A quantitative analysis was performed to compare the normalized spectral count in E1E2 and control conditions, with a T-test comparison. The proteins are sorted by significance (p-value < 0.05). The normalized spectral count is indicated for each biological independent triplicate, as well as the merge value. Of note, technical contaminants like serum albumin, keratins, and biotin-binding proteins such as pyruvate carboxylase, methyl-crotonyl CoA carboxylase, propionyl CoA carboxylase and acetyl CoA carboxylase were removed from the protein lists.

Supplementary Table 2 Most significantly enriched pathways from Broad Institute’s GSEA software. NES represents normalised enrichment score. FDR represents false discovery rate.
