# Supplementary figures and images for: The HCV Envelope Glycoprotein Down-Modulates NF-κB Signalling and Associates With Stimulation of the Host Endoplasmic Reticulum Stress Pathway
Source: Front Immunol. 2022 Mar 15;13:831695. doi: 10.3389/fimmu.2022.831695 (PMC8964954; doi:10.3389/fimmu.2022.831695)

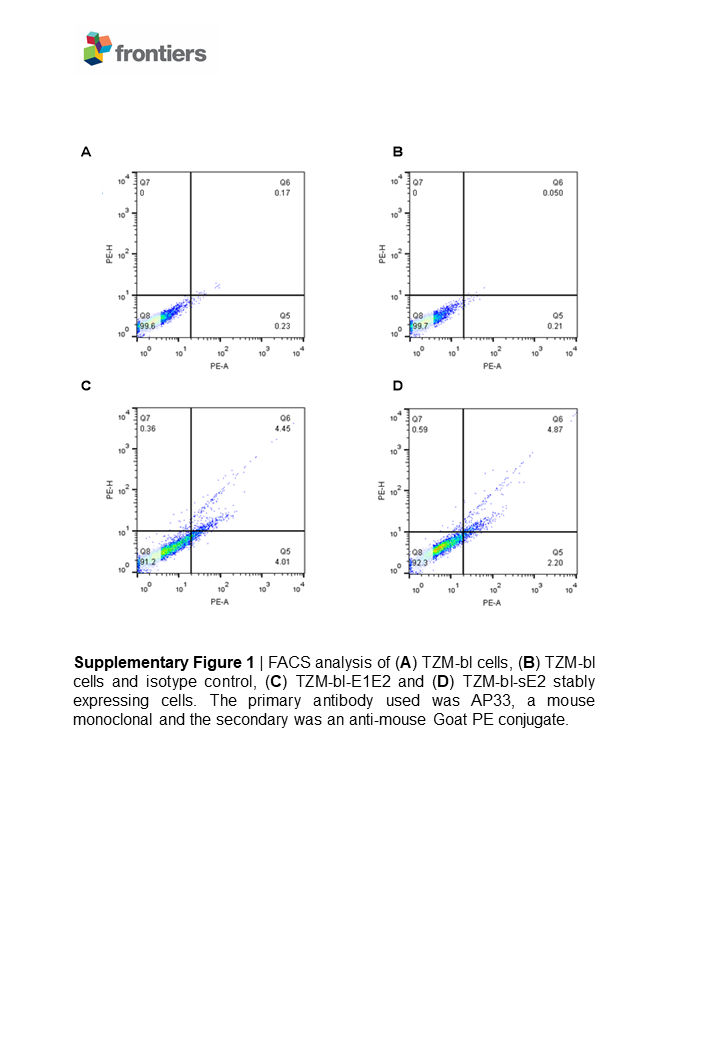

Supplement: Supplementary Figure 1 — FACS analysis of (A) TZM-bl cells, (B) TZM-bl cells and isotype control, (C) TZM-bl-E1E2 and (D) TZM-bl-sE2 stably expressing cells. The primary antibody used was AP33, a mouse monoclonal and the secondary was an anti-mouse Goat PE conjugate. [file Image_1.tif]

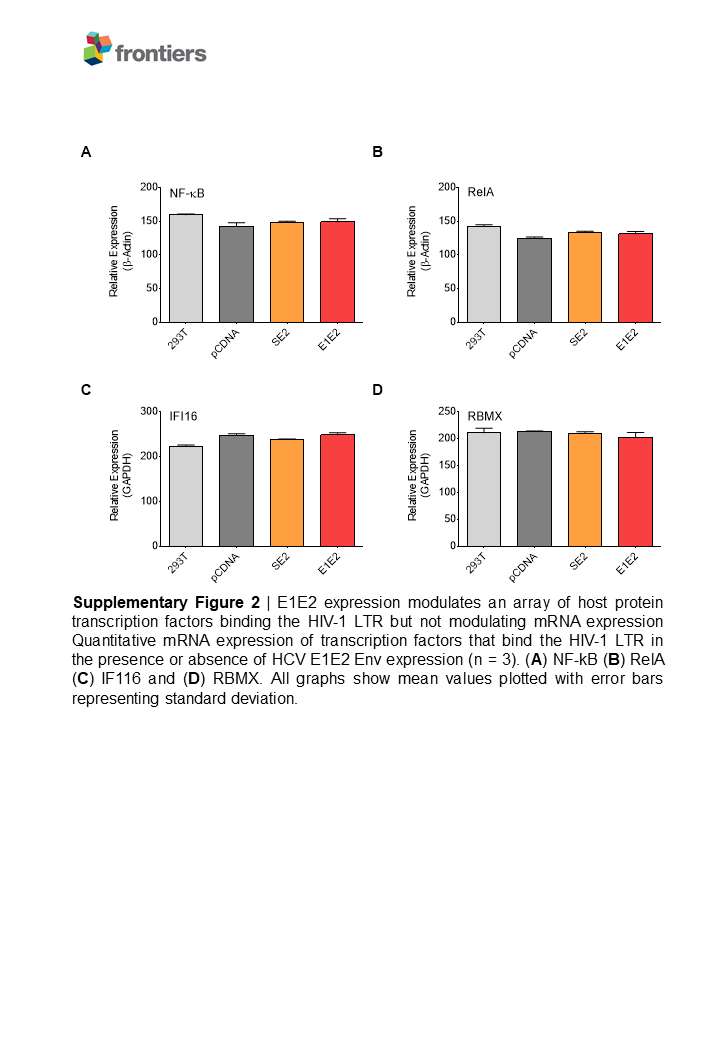

Supplement: Supplementary Figure 2 — E1E2 expression modulates an array of host protein transcription factors binding the HIV-1 LTR but not modulating mRNA expression Quantitative mRNA expression of transcription factors that bind the HIV-1 LTR in the presence or absence of HCV E1E2 Env expression (n = 3). (A) NF-κB (B) RelA (C) IF116 and (D) RBMX. All graphs show mean values plotted with error bars representing standard deviation. [file Image_2.tif]

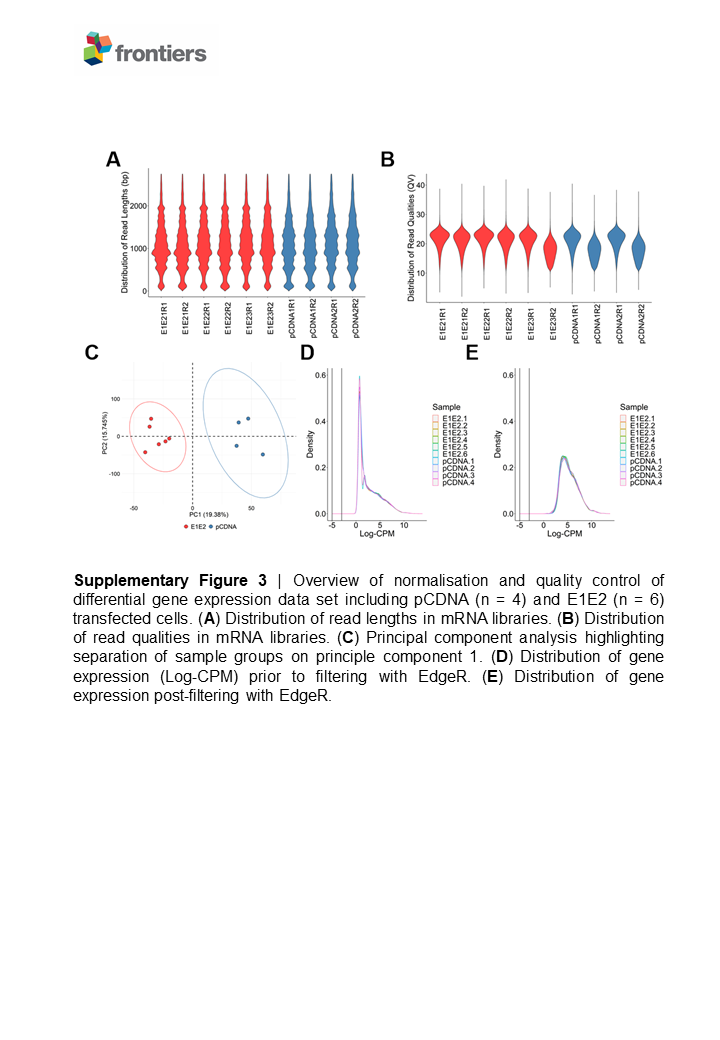

Supplement: Supplementary Figure 3 — Overview of normalisation and quality control of differential gene expression data set including pCDNA (n = 4) and E1E2 (n = 6) transfected cells. (A) Distribution of read lengths in mRNA libraries. (B) Distribution of read qualities in mRNA libraries. (C) Principal component analysis highlighting separation of sample groups on principle component 1. (D) Distribution of gene expression (Log-CPM) prior to filtering with EdgeR. (E) Distribution of gene expression post-filtering with EdgeR. [file Image_3.tif]

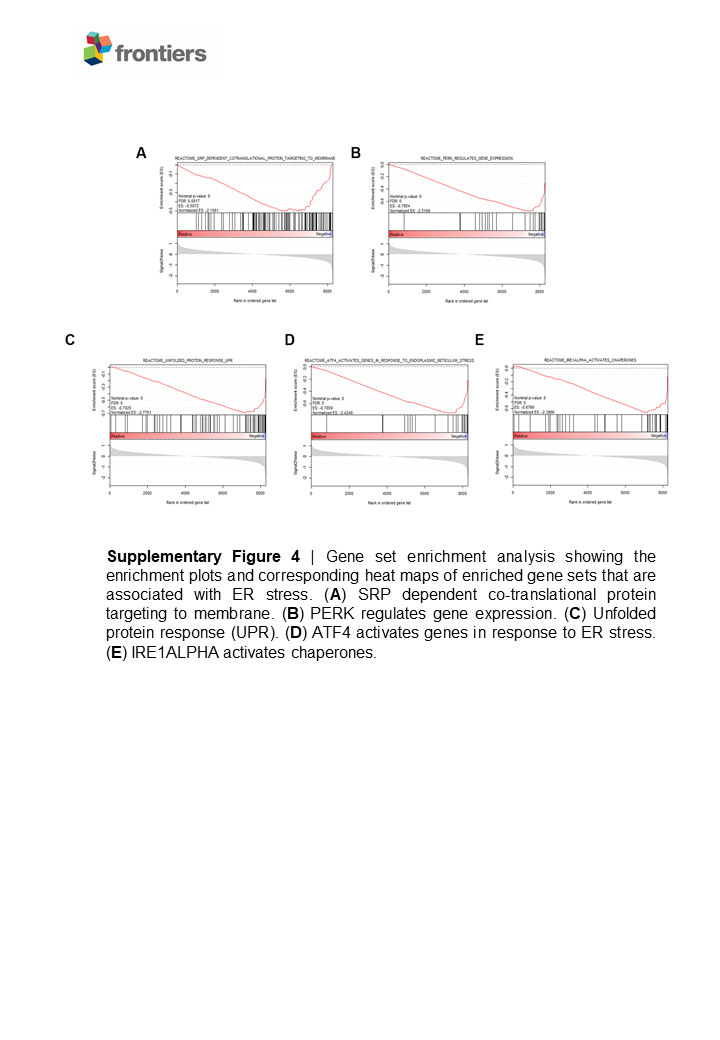

Supplement: Supplementary Figure 4 — Gene set enrichment analysis showing the enrichment plots and corresponding heat maps of enriched gene sets that are associated with ER stress. (A) SRP dependent co-translational protein targeting to membrane. (B) PERK regulates gene expression. (C) Unfolded protein response (UPR). (D) ATF4 activates genes in response to ER stress. (E) IRE1ALPHA activates chaperones. [file Image_4.tif]

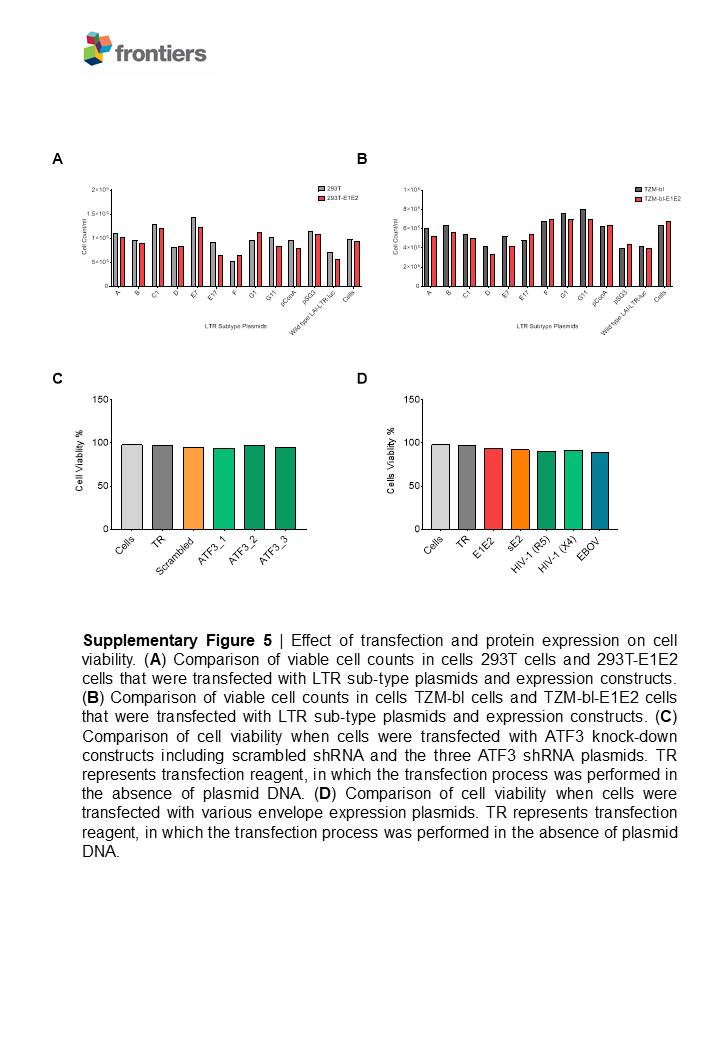

Supplement: Supplementary Figure 5 — Effect of transfection and protein expression on cell viability. (A) Comparison of viable cell counts in cells 293T cells and 293T-E1E2 cells that were transfected with LTR sub-type plasmids and expression constructs. (B) Comparison of viable cell counts in cells TZM-bl cells and TZM-bl-E1E2 cells that were transfected with LTR sub-type plasmids and expression constructs. (C) Comparison of cell viability when cells were transfected with ATF3 knock-down constructs including scrambled shRNA and the three ATF3 shRNA plasmids. TR represents transfection reagent, in which the transfection process was performed in the absence of plasmid DNA. (D) Comparison of cell viability when cells were transfected with various envelope expression plasmids. TR represents transfection reagent, in which the transfection process was performed in the absence of plasmid DNA. [file Image_5.tif]

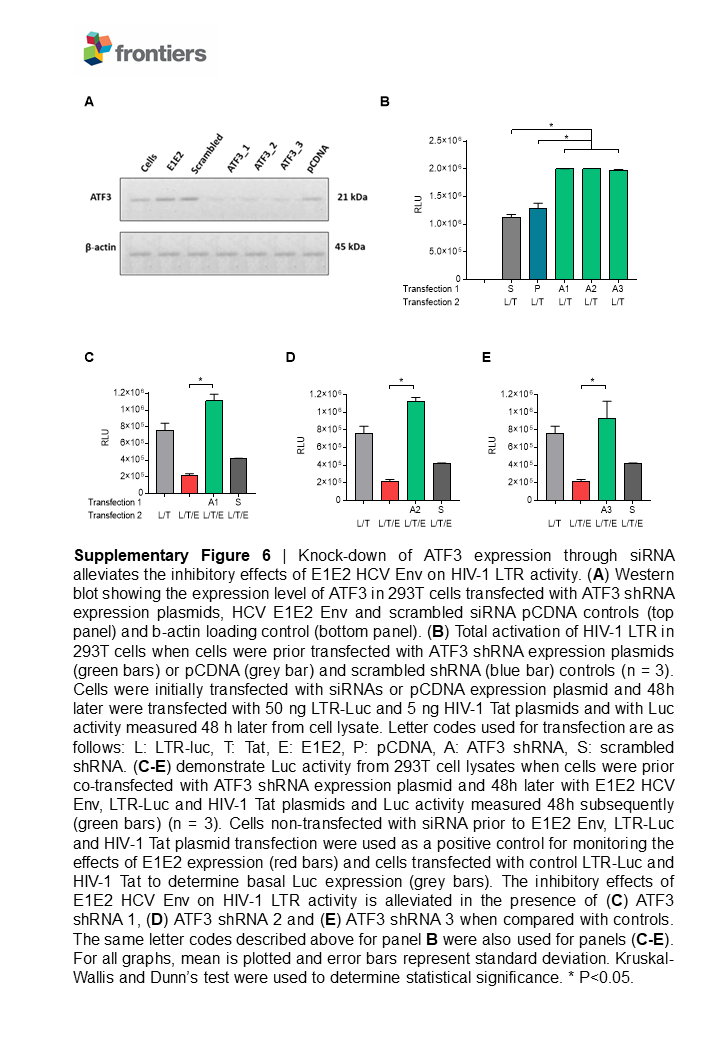

Supplement: Supplementary Figure 6 — Knock-down of ATF3 expression through siRNA alleviates the inhibitory effects of E1E2 HCV Env on HIV-1 LTR activity. (A) Western blot showing the expression level of ATF3 in 293T cells transfected with ATF3 shRNA expression plasmids, HCV E1E2 Env and scrambled siRNA pCDNA controls (top panel) and β-actin loading control (bottom panel). (B) Total activation of HIV-1 LTR in 293T cells when cells were prior transfected with ATF3 shRNA expression plasmids (green bars) or pCDNA (grey bar) and scrambled shRNA (blue bar) controls (n = 3). Cells were initially transfected with siRNAs or pCDNA expression plasmid and 48h later were transfected with 50 ng LTR-Luc and 5 ng HIV-1 Tat plasmids and with Luc activity measured 48 h later from cell lysate. Letter codes used for transfection are as follows: L: LTR-luc, T: Tat, E: E1E2, P: pCDNA, A: ATF3 shRNA, S: scrambled shRNA. (C–E) demonstrate Luc activity from 293T cell lysates when cells were prior co-transfected with ATF3 shRNA expression plasmid and 48h later with E1E2 HCV Env, LTR-Luc and HIV-1 Tat plasmids and Luc activity measured 48h subsequently (green bars) (n = 3). Cells non-transfected with siRNA prior to E1E2 Env, LTR-Luc and HIV-1 Tat plasmid transfection were used as a positive control for monitoring the effects of E1E2 expression (red bars) and cells transfected with control LTR-Luc and HIV-1 Tat to determine basal Luc expression (grey bars). The inhibitory effects of E1E2 HCV Env on HIV-1 LTR activity is alleviated in the presence of (C) ATF3 shRNA 1, (D) ATF3 shRNA 2 and (E) ATF3 shRNA 3 when compared with controls. The same letter codes described above for panel B were also used for panels (C–E). For all graphs, mean is plotted and error bars represent standard deviation. Kruskal-Wallis and Dunn’s test were used to determine statistical significance. * P<0.05. [file Image_6.tif]
