# Supplementary material for: The HCV Envelope Glycoprotein Down-Modulates NF-κB Signalling and Associates With Stimulation of the Host Endoplasmic Reticulum Stress Pathway
Source: Front Immunol. 2022 Mar 15;13:831695. doi: 10.3389/fimmu.2022.831695 (PMC8964954; doi:10.3389/fimmu.2022.831695)
Supplement: Supplementary Table 2 — Most significantly enriched pathways from Broad Institute’s GSEA software. NES represents normalised enrichment score. FDR represents false discovery rate. [file Table_2.docx]

| Pathway | NES | Enrichment | Nominal P-value | FDR | Function |
| --- | --- | --- | --- | --- | --- |
| REACTOME unfolded protein response (UPR) | -2.78 | Upregulated | 0.0000 | 0.0000 | Endoplasmic reticulum stress |
| REACTOME PERK regulates gene expression | -2.52 | Upregulated | 0.0000 | 0.0000 | Endoplasmic reticulum stress |
| REACTOME ATF4 activates genes in response to ER stress | -2.42 | Upregulated | 0.0000 | 0.0000 | Endoplasmic reticulum stress |
| REACTOME IRE1α activates chaperones | -2.39 | Upregulated | 0.0000 | 0.0000 | Endoplasmic reticulum stress |
| REACTOME SRP dependent cotranslational protein targeting to membrane | -2.16 | Upregulated | 0.0000 | 0.0017 | Endoplasmic reticulum signalling |
| KEGG protein export | -2.08 | Upregulated | 0.0005 | 0.0052 | Protein export |
| REACTOME response of EIF2AK4 GCN2 to amino acid deficiency | -2.01 | Upregulated | 0.0000 | 0.0127 | Cellular response to stress |
| REACTOME tristetraprolin (TTP) zfp36 binds and destabilizes mRNA | -1.99 | Upregulated | 0.0002 | 0.0146 | Regulation of mRNA stability |
| PID TGFBR pathway | -1.97 | Upregulated | 0.0007 | 0.0177 | TGF-β pathway |
| REACTOME butyrate response factor 1 (BRF1) binds and destabilizes mRNA | -1.96 | Upregulated | 0.0005 | 0.0169 | Regulation of mRNA stability |

**Supplementary Table 2** | Most significantly enriched pathways from Broad Institute’s GSEA software. NES represents normalised enrichment score. FDR represents false discovery rate.
